# Systematic Evaluation of Regiochemistry and Lipidation of Aryl Trehalose Mincle Agonists

**DOI:** 10.3390/ijms251810031

**Published:** 2024-09-18

**Authors:** Asia Marie S. Riel, Viktoria Rungelrath, Tamer A. Elwaie, Omer K. Rasheed, Linda Hicks, George Ettenger, Dai-Chi You, Mira Smith, Cassandra Buhl, Walid Abdelwahab, Shannon M. Miller, Alyson J. Smith, David Burkhart, Jay T. Evans, Kendal T. Ryter

**Affiliations:** 1 Department of Chemistry and Biochemistry, Center for Translational Medicine, University of Montana, Missoula, MT 59812, USA; asiamarie.riel@mso.umt.edu (A.M.S.R.); tamer.elwaie@mso.umt.edu (T.A.E.); omer.rasheed@inimmune.com (O.K.R.); dai-chi.you@mso.umt.edu (D.-C.Y.); 2 Department of Biomedical and Pharmaceutical Sciences, Center for Translational Medicine, University of Montana, Missoula, MT 59812, USA; viktoria.rungelrath@mso.umt.edu (V.R.); linda.hicks@mso.umt.edu (L.H.); miradevismith@gmail.com (M.S.); walid.abdelwahab@mso.umt.edu (W.A.); shannon.m.miller@inimmune.com (S.M.M.); smith.alysonj@gmail.com (A.J.S.); david.burkhart@mso.umt.edu (D.B.); jay.evans@mso.umt.edu (J.T.E.); 3 Department of Pharmaceutical Chemistry, Faculty of Pharmacy, Cairo University, Cairo 11562, Egypt; 4 Inimmune Corporation, Missoula, MT 59802, USA

**Keywords:** Mincle, agonist, trehalose, adjuvant

## Abstract

The Macrophage-Inducible C-type Lectin receptor (Mincle) plays a critical role in innate immune recognition and pathology, and therefore represents a promising target for vaccine adjuvants. Innovative trehalose-based Mincle agonists with improved pharmacology and potency may prove useful in the development of Th17-mediated adaptive immune responses. Herein, we report on in vitro and in silico investigations of specific Mincle ligand–receptor interactions required for the effective receptor engagement and activation of Th17-polarizing cytokines. Specifically, we employed a library of trehalose benzoate scaffolds, varying the degree of aryl lipidation and regiochemistry that produce inflammatory cytokines in a Mincle-dependent fashion. In vitro interleukin-6 (IL-6) cytokine production by human peripheral blood mononuclear cells (hPBMCs) indicated that the lipid regiochemistry is key to potency and maximum cytokine output, with the tri-substituted compounds inducing higher levels of IL-6 in hPBMCs than the di-substituted derivatives. Additionally, IL-6 production trended higher after stimulation with compounds that contained lipids ranging from five to eight carbons long, compared to shorter (below five) or longer (above eight) carbon chains, across all the substitution patterns. An analysis of the additional cytokines produced by hPBMCs revealed that compound **4d**, tri-substituted and five carbons long, induced significantly greater levels of interleukin-1β (IL-1β), tumor necrosis factor- α (TNF-α), interleukin-23 (IL-23), and interferon- γ (IFN-γ) than the other compounds tested in this study. An in silico assessment of **4d** highlighted the capability of this analogue to bind to the human Mincle carbohydrate recognition domain (CRD) efficiently. Together, these data highlight important structure–activity findings regarding Mincle-specific cytokine induction, generating a lead adjuvant candidate for future formulations and immunological evaluations.

## 1. Introduction

The Macrophage-Inducible C-Type Lectin receptor (Mincle) has gained traction in the past decade as a potential vaccine adjuvant target, as it can influence innate immune responses during infection, inflammation, and autoimmunity [1,2,3]. Mincle is highly expressed on all subsets of macrophages, and its activation can initiate pro-inflammatory cytokines (i.e., IL-6, IL-1β, IL-23, and TNF-α), which can induce the polarization of T helper cells towards a Th17 phenotype [1]. As a pattern-recognition C-Type Lectin receptor (CLR), Mincle contains a calcium-dependent carbohydrate recognition domain (CRD), in which ligand-bound activation leads to the inflammatory responses critical to immune stimulation [4,5]. Mincle primarily recognizes glycolipids, such as α-Mannose [3], Glyceroglycolipids [6], and other fungal and bacterial glycolipids, making it an attractive adjuvant and immunotherapy target [7]. Evidence is mounting that the synergistic Th1 and Th17 adaptive immune responses are necessary for protection against bacterial and fungal pathogens, such as *Mycobacterium tuberculosis* and *Candida albicans* [8,9]. However, to date, there are no approved adjuvants for stimulating a robust Th17 response in humans [10], underscoring the need for innovative Th17-inducing compounds.

**Figure 1:**
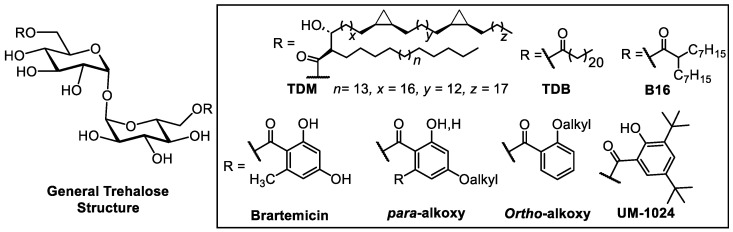
Representative Mincle ligands TDM, TDB, and Brartemicin, and previously published Mincle ligands.

The natural mycobacterial cell wall component cord factor, 6,6′-trehalose dimycolate (TDM) [11,12], and its synthetic glycolipid analogues, 6,6′-trehalose dibehenate (TDB, Figure 1) [12] and trehalose dicorynomycolate, can be recognized by Mincle. However, their high lipophilic nature and poor solubility require creative formulation efforts and restricting their formulation versatility. For example, TDB/dimethyldioctadecylammonium bromide (DDA) has been pursued clinically as Cationic Adjuvant Formulation 01 (CAF01), and exhibits IL-17 (Th17 effector cytokine) production in mice [5,13]. However, no robust Th17 responses have been reported in humans, despite long-lasting Th1 immunity [14]. Alternative Mincle agonists, based on trehalose diester glycolipid analogs, have been explored as Th17-inducing adjuvants [12,15,16].

The aryl trehalose benzoate natural product, Brartemicin, 6,6′-bis(2,4dihydroxy-6-methylbenzoyl)-α,α-D-trehalose (Figure 1), was described as having a high-affinity ligand for the Mincle CRD, exhibiting a 290-fold increase in receptor affinity compared to α,α-trehalose [17]. However, Brartemicin leads to minimal signaling activity in mouse RAW cells and human peripheral blood mononuclear cells (hPBMCs) [17,18]. Notably, computations have highlighted the favorable interaction of the benzoate group with the proximal hydrophobic groove adjacent to the bovine Mincle CRD [17]. This insight inspired the exploration of benzoate derivatives as a potential avenue for enhancing the activity and affinity of Mincle agonists. The design and synthesis of innovative trehalose-based Mincle agonists is necessary to expand our knowledge of the complex receptor–ligand interactions that can induce Mincle receptor signaling and elicit Th17-polarizing adjuvant activity.

Several recent reports have demonstrated the novelty and biological impact of benzoate trehalose-derived Mincle agonists, and have begun to explore the specificity of the observed activity relative to aryl substitution and the degree of lipidation through computational, in vitro, and in vivo investigations [18,19,20,21,22,23]. The structural requirements for Mincle activation have yet to be fully investigated, and we also need a better understanding of how these ligands not only induce cytokines via Mincle, but also the impact of the structure–ligand interactions on the in vivo Th17 response [24]. Stoker et al. demonstrated that *o*-, *p*-, and *m*,*m*-substitution of lipidated benzoate analogs were potent Mincle agonists, with *o*- and *m*,*m*-substitution exhibiting desirable inflammatory responses in mice in in vitro and in vivo experiments (see some examples in Figure 1) [19,20,21]. However, these highly lipidated analogues were less active in human cells in vitro. To optimize the activity of this class of ligands, we envisioned a systematic structural investigation of lipidation and aryl substitution to achieve higher potency and adjuvant activity in humans. To date, Mincle agonists have been identified within two major classes: highly lipidated alkyl glycosides and benzoyl/benzamide trehalose analogues, such as UM-1024 (Figure 1) [18,19,20,21,22,23]. The initial libraries of lipidated benzoyl trehalose analogues [18,19,20,21,22,23] have suggested that the regiochemistry of alkoxy benzoyl derivatives is critical to their activity. This prompted the development of an expanded compound library in this study, enabling the systematic exploration and optimization of Mincle activity, with the aim of improving the pharmacological properties of the ligands. It was hypothesized that degree of lipidation may circumvent the need for long lipid chains, allowing for easier-to-formulate drug-like adjuvants for use in human clinical vaccine applications. Herein, we describe the systematic evaluation of the regiochemistry and degree of lipidation of aryl trehalose analogues, generating a lead adjuvant candidate with dramatically improved potency and potential for human clinical application.

## 2. Results

### 2.1. Design and Synthesis of 6,6′-Diaryl Trehalose Analogues

**Figure 2:**
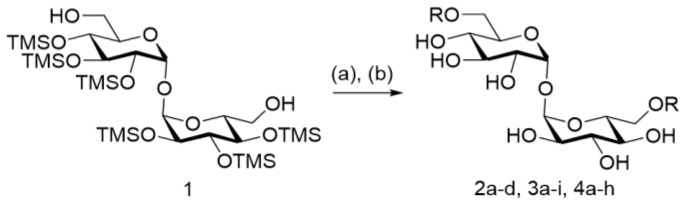
Synthesis of 6,6′-diaryl trehalose derivatives prepared from readily accessible 1. Reagents and conditions: (**a**) aryl acid, DCC or EDC-MeI, 4-(dimethylamino)pyridinium *p*-toluenesulfonate or DMAP, and DCM; (**b**) Dowex 50W8X, Tetra-*n*-butylammonium fluoride (1.0 M in THF) or 3N HCl in MeOH, and DCM or THF.

To fully elucidate the effect of lipid length and the substitution pattern of compounds, we prepared a focused library with an array of alkoxy lipid lengths from C_1_ to C_10_ with three different substitution patterns: 3,4-, 3,5-, and 3,4,5-alkoxy benzoate derivatives, based on the optimal length of the lipid described for trehalose diesters (TDEs) [18] (Figure 2). The alkoxy benzoic acids were synthesized from commercially available methyl 3,4-dihydroxybenzoate, methyl 3,5-dihydroxybenozate, or methyl 3,4,5-trihydroxybenzoate, respectively, via potassium hydroxide and tetra-n-butylammonium bromide-mediated ether formation, with the respective alkyl bromide followed by ester hydrolysis. The synthetically accessible 6,6′-dihydroxyhexasilyl trehalose **1** was modified with the appropriate aryl carboxylic acid using our standard protocol, using either 1-ethyl-3-(3-(dimethylamino)propyl)carbodiimide methiodide (EDC-MeI) and dimethylaminopyridine (DMAP) or dicyclohexyl carbodiimide (DCC), in the presence of *N*,*N*-dimethyl-4-pyridinaminium 4-methylbenzenesulfonate. The subsequent global silyl deprotection under acidic conditions provided the respective TDE. Each TDE derivative shown in Table 1 was isolated by chromatography and its purity was determined by LC-MS to be at least 95%. Structural confirmation was determined by ^1^H, ^13^C NMR, and HRMS (see Supplementary Materials for Figures S1–S18 for chemical characterization data).

### 2.2. IL-6 Response of hPBMCs to Stimulation with Aryl Trehalose Glycolipids

**Figure 3:**
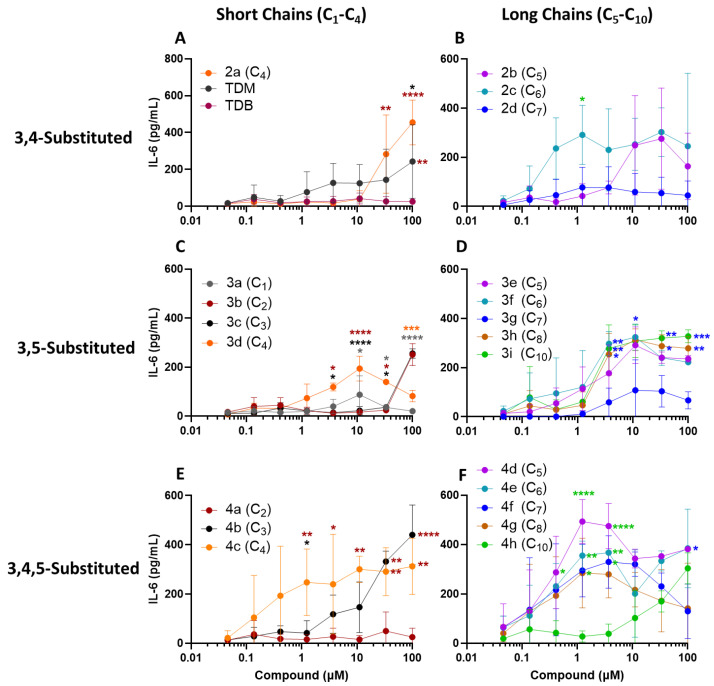
IL-6 production of hPBMCs in response to stimulation with synthetic trehalose diester compounds. The derivatives are separated by substitution pattern and chain length. (**A**,**C**,**E**) are the short-chained C_1_ to C_4_, respective of their substitution. (**B**,**D**,**F**) are the mid-to-long-chained C_5_ to C_10_, respective of their substitution. The compounds were dissolved in EtOH, serially diluted in EtOH, and then dried onto the bottom of a tissue culture plate. The fresh hPBMCs were isolated and added to the compound-coated plates and incubated at 37 °C for 24 h. The supernatants were harvested and analyzed for IL-6 via ELISA (*n* = 3 individual donors). The data are displayed as the mean plus SD and were analyzed for statistical significance using a two-way ANOVA with Tukey’s multiple comparisons test. The significances were considered as follows: * *p* ≤ 0.05, ** *p* ≤ 0.01, *** *p* ≤ 0.001, and **** *p* ≤ 0.0001. Significant differences between compounds are indicated at the given concentrations. The color of the asterisk above or to the right of a data point indicates to which color-matched compound a significant difference was measured.

For the purpose of developing a human active Mincle agonist, the compounds described in Table 1 were tested for their activation of human PBMCs, as measured through cytokine production. Mincle activation of hPBMCs is known to elicit the induction of inflammatory cytokines TNF-α, IL-6, IL-1β, and IL-23 [1,5,6]. The utilization of human PBMCs in the evaluation of agonists as effective Mincle adjuvants has been described previously [18]. To assess the immunostimulatory activity and evaluate the structure–activity relationships between alkyl chain length and regiochemistry, all the compounds were compared head-to-head using plate deposition, from an ethanol stock solution, measuring the induction of a Th17-polarizing innate cytokine, IL-6 (Figure 3); the control molecules for Mincle activation included TDB and TDM.

To aid the comparative analysis, the compounds were grouped according to their substitution pattern and chain length. The short-chained derivatives were considered those one to four (C_1_–C_4_) carbons long, and the mid-to-long-chained derivatives were five to ten (C_5_–C_10_) carbons long. With regard to the impact of the carbon chain length on cytokine production, the long-chain compounds (Figure 3, right column) trended toward the induction of higher levels of IL-6 at lower concentrations (i.e., <100 µM) than the short-chain compounds (Figure 3, left column) across all the substitution patterns. However, several exceptions from this pattern were noted, with **2d** (C_7_), **3g** (C_7_), and **4h** (C_10_) showing lower activity than the other long-chain compounds with their respective substitution pattern, and **4c** (C_4_) showing higher activity relative to the other short-chain compounds.

Within the 3,4-substitution pattern, **2c** (C_6_) induced higher levels of IL-6 than **2b** (C_5_) and **2d** (C_7_) at a dose range of 0.5–5µM, but the difference was only significant at 1.2 µM (Figure 3B). On the short-chain side of the 3,4-substitution pattern, **2a** (C_4_) induced significantly higher IL-6 levels than TDB at 33–100µM, and significantly higher IL-6 than TDM at 100 µM (Figure 3A).

Interestingly, within the 3,5-substitution pattern, the C_5_–C_10_ length compounds induced similar levels of IL-6 over a wide dose range, with the exception of the C_7_ derivative **3g**. In fact, **3g** induced significantly less IL-6 than the other long-chain compounds in the same substitution pattern, for a dose range of 3.7–100 µM (Figure 3D). On the short-chain side, and similar to the 3,4-substituted compounds, the C_4_ derivative **3d** induced significantly higher levels of IL-6 than the shorter-chain compounds **3a**, **3b**, and **3c** at 11.1 and 33.3 µM, but not at higher doses (Figure 3C).

Lastly, regarding the 3,4,5-substituted compounds, it was found that the short-chained C_3_–C_4_ compounds **4b** and **4c** trended toward greater IL-6 induction at relatively lower concentrations than the compounds with an equal chain length but different substitution patterns (Figure 3, left column). Compound **4a** (C_2_) induced significantly less IL-6 than **4b** (C_3_) and **4c** (C_4_) at the multiple concentrations tested (Figure 3E). This matches the low IL-6-inducing activity of the C_2_ compound **3b** in the 3,5-substitution pattern (Figure 3C). All the tri-substituted compounds with mid-to-long chain lengths (Figure 3F) induced maximal IL-6 responses for a concentration range of 1–5 µM, with the exception of the C_10_ compound **4h**. In fact **4d**, **4e**, and **4f** induced significantly more IL-6 than **4h** at a concentration range of 1.2–3.7 µM. Of note, the C_5_ compound **4d** was found to not only induce the highest maximal IL-6 production (493 pg/mL) compared to all the other tested compounds, but also to induce it at the low dose of 1.2 µM.

In summary, both the chain length and the substitution pattern were found to influence the IL-6 responses of the hPBMCs. The mid chain lengths (C_5_–C_8_) trended toward maximal IL-6 responses at lower concentration ranges than the shorter (C_1_–C_4_) and longer (C_8_–C_10_) chain length compounds. Furthermore, the 3,4,5-substituted compounds trended toward more activity of IL-6 at lower doses than the di-substituted derivatives.

### 2.3. Evaluation of an Expanded Cytokine Profile of Lead Mincle Agonists

In addition to IL-6, other cytokines have been implicated in the polarization of Th17 and Th1 T cells downstream of Mincle activation [1,2,3,4,5]. Therefore, additional cytokines were measured after stimulation with the chosen aryl trehalose glycolipids. As the longer chain-length compounds trended toward more activity than the shorter-chain compounds with IL-6 as the readout, only the lead di- and tri-substituted C_5_ through C_8_ trehalose derivatives were investigated; specifically, we tested their potential to induce the additional cytokines, IL-1β, IL-23, INF-γ, and TNF-α. IL-6 was measured again to confirm the previous figures (Figure S19). The cytokines were measured using a multiplex Mesocale Discovery U-PLEX Assay (MSD) [25].

**Figure 4:**
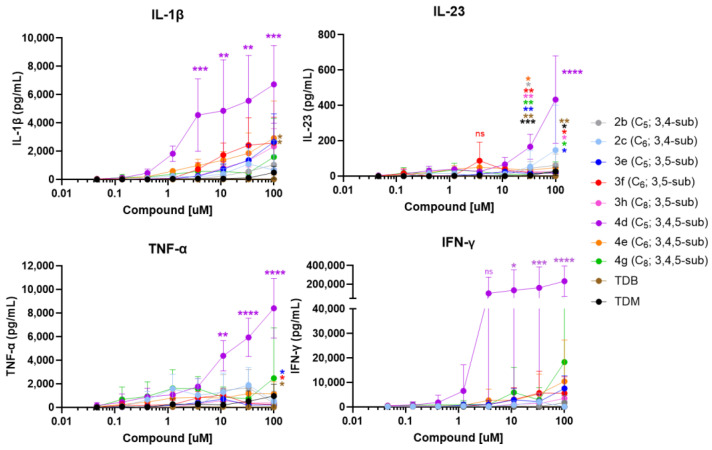
Results using MesoScale Discovery multiplex cytokine assay to measure IL-1β, IL-23, INF-γ, and TNF-α cytokine production by human PBMCs in response to stimulation with eight trehalose diester compounds. The compounds were dissolved in EtOH, serially diluted in EtOH, and then dried to the bottom of a tissue culture plate. The fresh hPBMCs were isolated and added to the compound-coated plates and incubated at 37 °C for 24 h. The supernatants were harvested and analyzed for IL-1β, IL-23, INF-γ, and TNF-α via MesoScale Discovery multiplex cytokine assay (*n* = 3 individual donors). The data were analyzed for statistical significance using a two-way ANOVA with Tukey’s multiple comparisons test. The significances were considered as follows: ns = *p* > 0.05, * *p* ≤ 0.05, ** *p* ≤ 0.01, *** *p* ≤ 0.001, and **** *p* ≤ 0.0001. Significant differences between compounds are indicated at the given concentrations. The color of the asterisk above or to the right of a data point indicates to which color-matched compound a significant difference was measured. For IL-1β, TFN-α, IL-23, and IFN-γ, the purple asterisks indicate the significant difference of 4d to all other the compounds at the given concentration. If the significant difference between 4d and the other compounds varied, the asterisk indicating the biggest *p* value was graphed.

Similar to the previous experiment (Figure 3), all the compounds tested induced IL-6 secretion from the hPBMCs, except TDB (Figure S19). The tri-substituted compounds **4d**, **4e**, and **4g** induced higher levels of IL-6 at 1 µM than the 3,5-substituted compounds (**3e**, **3f**, and **3h**), and the difference was significant for **4d** over all the 3,5-subsituted compounds, and for **4e** and **4g** over the 3,5-substituted **3h**. Additionally, the tri-substituted **4d** induced significantly more IL-6 at both 1.2 and 100 µM than the 3,4-substituted **2b**, but not the 3,4-substituted **2c** (Figure S19), thus showing a similar trend to the previous experiment (Figure 3B). Interestingly, **4d** induced significantly more IL-1β, TNF-α, IL-23, and IFN-γ than all the other compounds at the indicated concentrations. Furthermore, **4g**, also a tri-substituted compound, induced significantly higher TNF-α than the 3,5-substituted **3f** and **3h** at 100 µM (Figure 4).

In summary, the analysis of an extended spectrum of cytokines revealed that the 3,4,5-substituted C_5_ compound **4d** induced significantly greater levels of IL-1β, TNF-α, IL-23, and IFN-γ than the compounds with an equal chain length within the different substitution patterns. These results held steadfast among the compounds with the same tri-substitution pattern, as alternate chain lengths exhibited diminished cytokine levels.

### 2.4. In Silico Evaluation of Substitution Pattern of Mincle Ligands

The differences in activity observed for the compounds reported here suggest that the structural variations among the ligands affect their binding to the Mincle CRD. To gain further insight into the mode of binding, we used molecular docking to better elucidate their interaction complex. As the substitution pattern tended to have more of an effect on cytokine production than the chain length, we performed molecular docking studies using the Cresset Flare Software Suite (Version 8), to confirm and analyze the binding characteristics of the C_5_-lipidated derivatives for each substitution pattern (**2b**, **3e**, and **4d**), within the CRD of human Mincle (PDB: 3WH2). The Mincle literature commonly uses the bovine Mincle CRD for molecular docking, as it is the only available Mincle CRD crystal structure co-crystalized with trehalose. Therefore, bovine Mincle (PDB: 4KZV) is a critical computational starting point for determining a potential α,α-trehalose-specific recognition domain within the CRD of human Mincle with a Glu-Pro-Asn (EPN) motif. Accordingly, α,α-trehalose was subjected to a molecular docking analysis within the binding pocket of human Mincle CRD (PDB: 3WH2) [26,27]. This investigation aimed to delineate the putative binding domain and interaction dynamics of α,α-trehalose with human Mincle CRD, informing subsequent docking studies involving new investigational chemical entities.

**Figure 5:**
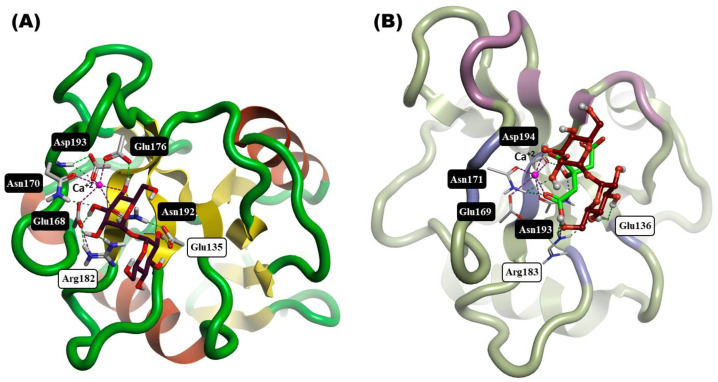
Bovine and human Mincle CRD computational comparison and evaluation. (**A**) The interaction of the co-crystallized α,α-trehalose (maroon sticks) inside the CRD of bovine Mincle (PDB: 4KZV), with the ribbon representation colored by secondary structure. (**B**) The alignment between the co-crystallized citrate (green sticks) and the docked pose of α,α-trehalose (maroon ball & sticks) in human Mincle CRD (PDB: 3WH2), where the ribbon is colored by protein chain. Dashed green lines indicate hydrogen bonds, and pink dashed lines denote salt bridge interactions (Ca chelation).

The primary binding site of bovine Mincle at the conserved Ca^2+^ CRD binding site shows the five canonical amino acids (Glu168, Asn170, Glu176, Asn192, and Asp193) interacting with the Ca^2+^ ion (Figure 5A black boxes). Four of these amino acids are involved in the primary interaction with the 3- and 4-hydroxyl groups of one glucose residue, while Glu135 and Arg182 form hydrogen bonds with the 2-hydroxyl of the other glucose unit. Similarly, the molecular docking of α,α-trehalose into human Mincle CRD demonstrates the capability of the docking pose to recapitulate the critical interactions observed in the co-crystal structure of α,α-trehalose bound to bovine Mincle, specifically involving the residues within the CRD and calcium ion coordination (Figure 5A,B). This supports the suitability of this model (Figure 5B) as a reliable framework for exploring the molecular interactions in silico in the current investigation.

**Figure 6:**
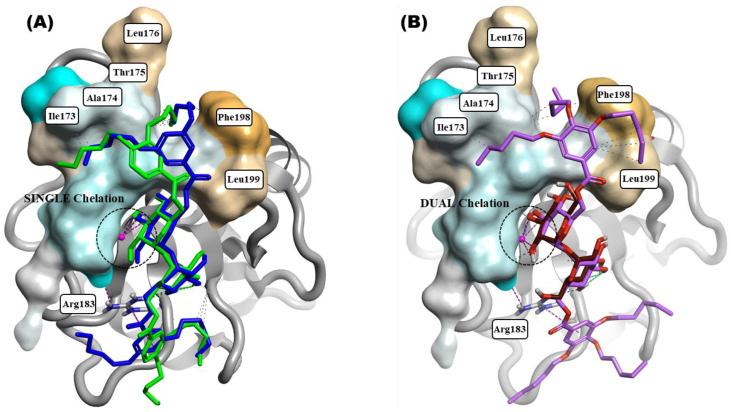
In silico model of interactions for all three tri-substituted derivatives. Docking pose alignment of **3e** ((**A**), blue sticks) and **2b** ((**A**), green sticks), highlighting single Ca^+2^ chelation and differential hydrophobic groove interaction with emphasis on reduced binding affinity in **2b** compared to **3e**. Docking pose of **4d** ((**B**), violet sticks) aligned with α,α-trehalose (maroon sticks), demonstrating dual Ca^+2^ chelation and optimal hydrophobic groove binding in hMincle (PDB: 3WH2), highlighting tri-alkoxy substitution and surface hydrophobicity mapping. Dashed lines represent different interactions: green for H-bonds, grey for hydrophobic contacts, and pink for cation-π stacking/metal chelation. Surface color representation: cyan indicates hydrophilic regions and brown denotes hydrophobic regions.

The docked compounds (**2b**, **3e**, and **4d** shown in Figure 6) exhibited similar binding patterns to the first glucose residue of α,α-trehalose within the CRD of Mincle, and chelate Ca^2+^ with at least one hydroxyl group, with a predicted high affinity and docking LigandFit Volume Score (LF VScore) ranging from −13.80 to −11.78. Interestingly, the second glucose residue in all the docked compounds revealed strong interactions at the secondary binding site of the CRD, and showed two hydrogen bonds with Glu136 and at least one cation–π interaction with the Arg183 amino acid residue. Noteworthy, the interaction with Arg183 is implicated as crucial for activity, as previously described in [21].

Performing a closer examination (Figure 6) of the substitution pattern in silico binding LF VScore within the CRD, the tri-substituted five-carbon derivative **4d** exhibited a −13.80 while, in contrast, the positional isomers **3e** (3,5-substituted C_5_) and **2b** (3,4-substituted C_5_) demonstrated LF VScore values of −12.56 and −11.78, respectively. As depicted in Figure 6B, **4d** exhibits a distinct capability for binding within the hydrophobic groove of hMincle CRD. Specifically, **4d** engages in multiple hydrophobic and π-π interactions with key amino acid residues lining the groove, including Ile173, Ala174, Thr175, Phe198, and Leu199. These interactions are crucial, as they stabilize the complex formed between **4d** and the Mincle CRD in this docking model. Moreover, the unique structural features of **4d** enable it to efficiently coordinate calcium ions through both the 3- and 4-hydroxyl groups of one glucose residue. The positional isomers **2b** (3,4-substituted C_5_) and **3e** (3,5-substituted C_5_) demonstrate differing binding affinities within the CRD or calcium chelation site of human Mincle, each via a single hydroxyl group. Furthermore, one of the alkoxy groups of **3e** (3,5-substituted C_5_) effectively occupies the hydrophobic groove, establishing robust hydrophobic interactions with key amino acid residues, such as Ala174, Thr175, and Phe198. In contrast, the positional isomer **2b** (3,4-substituted C_5_) struggles to fit into the hydrophobic groove, lacking an interaction with Phe198 and displaying only modest hydrophobic interactions with Ala174 and Thr175 (Figure 6A).

Given the exceptional ability of the tri-substituted C_5_-lipidated derivative **4d** to efficiently bind to hMincle and induce the highest levels of cytokines in the expanded profile compared to the other compounds in this study, it was crucial to further investigate the molecular interactions and dynamic behavior of **4d** when bound to hMincle. Consequently, molecular dynamics simulations (MDS) were performed on the docked pose complex of hMincle with compound **4d**, using the MDS platform in Cresset Flare. This approach was aimed at enhancing confidence in the accuracy of the docking results and the stability of the predicted binding pose, while also examining the dynamic changes in the conformation of the protein–ligand complexes.

In this study, it was noted that the potential energy of the **4d**–hMincle complex remained relatively constant throughout a 100 nanosecond (ns) simulation period, suggesting that the system reached a state of equilibrium (see Supplementary Materials, Figure S20). We evaluated the conformation changes in the hMincle–**4d** complex over the 100 ns simulation. The Root Mean Square Deviation (RMSD) was calculated throughout the simulation trajectory to assess the average displacement of frames relative to the initial frame.

**Figure 7:**
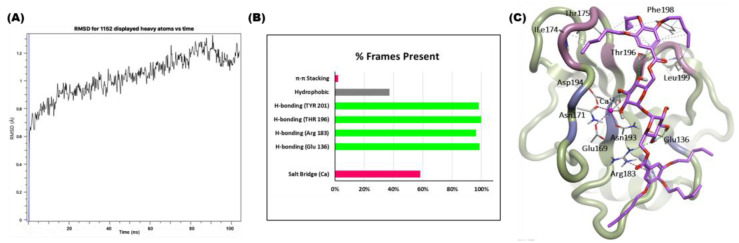
Structural analysis of the MDS trajectories. (**A**) The RMSD plot during 100 ns MDS, computed between the initial frame of the protein–ligand complex and the current frame over time. (**B**) A breakdown of the classical interactions during the 100 ns MD simulation. (**C**) The predicted 3D ligand interaction diagram of **4d** bound to hMincle.

The RMSD plot for the hMincle (3WH2) complex with **4d**, shown in Figure 7A, illustrates that the initial conformational fluctuations observed in the complex during the early part of the 100 ns simulation phase eventually stabilized during the subsequent production phase. In the RMSD plot, the hMincle–**4d** complex was stable, reaching equilibrium within the first few nanoseconds of the simulation time, with an average RMSD of 1.028 Å and a maximum of 1.328 Å. To evaluate the binding stability between compound **4d** and hMincle, we investigated the intermolecular interactions, including the hydrogen bonds (H-bonds), salt bridges (Ca-chelation), hydrophobic interactions, and π-π stacking, using 10,000 frames generated from a 100 ns molecular dynamics (MDs) trajectory. The interaction breakdown presented in Figure 7B indicates that **4d** engages in all available interaction types with binding site residues. According to the contact percentage plot (Figure 7B), **4d** establishes relatively stable direct Ca-chelation (salt bridge) (60%) and H-bond interactions, which remain highly stable throughout the simulation (96.50–99.80%). Conversely, the π-π stacking and hydrophobic interactions exhibit the lowest contact percentages (40%), likely due to the high flexibility of the C_5_-aliphatic chains. The minimal deviations of trehalose moiety in the CRD, which maintained the highest percentage of H-bonding contacts throughout the simulation, support the accuracy and reliability of the docking pose of **4d** in this in silico study. Taken together, the molecular docking and the MDS highlight the complementarity between **4d** and the Mincle CRD binding site.

## 3. Discussion

The main objective of this study was to identify and optimize a synthetic Mincle agonist through structure–activity relationship (SAR) studies. The identification of a novel optimized Mincle ligand could aid in the development of a vaccine adjuvant that could promote Th17 or Th1/17 immunity. To achieve this, we evaluated the Th17-polarizing and additional inflammatory cytokines produced by human PBMCs following stimulation with Mincle-targeting ligands. Together, these data highlight two important details about Mincle-mediated IL-6 induction: (1) the ideal aliphatic chain length range for the activation of hPBMCs appears to be five to eight carbons, and (2) the regiochemistry of the aryl ring trends toward 3,4,5-substitution. Interestingly, the addition of the third alkoxy group in the 3,4,5-substitution pattern seems to play a key role in the compounds’ ability to activate Mincle, as the maximal IL-6 levels were induced at lower concentrations compared to the di-substituted compounds of equal chain length. This work expands upon the previously reported regiochemical investigations [28], as we hypothesize that the tri-substituted aryl moiety is critical for Mincle activation in this scaffold. We hypothesize the increased binding affinity of the third alkoxy group to the Mincle CRD, possibly via the multiple hydrophobic and π-π interactions with the key amino acid residues identified through the in silico docking to human Mincle.

The compounds with chain lengths of C_5_–C_8_ exhibited the highest IL-6 response in the hPBMCs (Figure 3). The lack of response to stimulation with **4h** (C_10_) is also intriguing, as it appears that increasing the chain length beyond 10 carbons reduces IL-6 levels. Based on this finding, only medium-to-long carbon chain compounds **2b**, **2c**, **3e**, **3f**, **3h**, **4d**, **4e**, and **4g** were further investigated as potential lead adjuvants able to induce a combined synergistic Th1/Th17 cytokine response. Notably, **4d** (3,4,5-substituted C_5_) exhibited the induction of all three Th17-polarizing cytokines IL-6, IL-23, and IL-1β, as well as TNF-α and IFN-γ, compared to the other seven compounds shown in Figure 4. Additionally, **4d** induced significantly higher levels of cytokines than the benchmark adjuvants TDB and TDM.

The mode of binding of compound **4d** to the Mincle CRD is highly correlated with cytokine production, as shown in Figure 4. The binding affinity of **4d** can be attributed to its tri-substituted structure, which facilitates its optimal accommodation within the hydrophobic environment of the groove shown in Figure 6. This compound’s ability to dual chelate Ca^2+^ further enhances the computational binding affinity and stability within the binding site, distinguishing it from other derivatives, like **3e** (3,5-substituted C_5_) and **2b** (3,4-substituted C_5_), which predominantly interact via single hydroxyl group chelation (Figure 6A). This disparity likely contributes to the activity differences observed with **3e** and **2b**. Additionally, the MDSs allowed for a closer examination of the interactions between our lead molecule **4d** and the Mincle CRD. This study highlights the complementarity of the hydrogen bonding and calcium interactions of **4d** with Mincle.

In summary, the distinct binding of **4d** to human Mincle underscores its structural plasticity and specific interactions with critical residues, highlighting its potential as a promising candidate for further study and development in therapeutic applications targeting Mincle-mediated processes. IL-6 production by hPBMCs was used as an initial screening tool with which to differentiate the substitution and lipidation patterns on aryl trehalose-based Mincle agonists. Expanding the cytokine array to additional Th17-polarizing and Th1 cytokines enabled the identification of **4d** as having great potential as a Th1/Th17-inducing vaccine adjuvant.

## 4. Materials and Methods

### 4.1. General Experimental Procedure

All reagents and solvents were used as received. Reactions were monitored by TLC analysis on Merck Silica gel 60 F254 plates, visualized by UV at 254 nm and dipping in vanillin (vanillin/water/ethanol/sulfuric acid, 0.2 g:5 mL:5 mL:1 mL) or phosphomolybdic acid in ethanol (PMA), and developed with heat. All compounds were confirmed to be >95% pure by NMR and HPLC-DAD analysis. ^1^H and ^13^C NMR spectra were recorded on Agilent (Agilent Technologies, Santa Clara, CA, USA) or Bruker (Bruker, Billerica, MA, USA) 400 MHz instruments and were referenced to TMS or solvent peak. High-resolution HPLC-MS analysis was obtained using Agilent 6520 Q-TOF mass spectrometer (Agilent Technologies, Santa Clara, CA, USA) utilizing electrospray ionization source in positive or negative mode. Chromatography was performed on Buchi Grace (Buchi Corporation, New Castle, DE, USA) or Biotage Isolera One (Biotage, Uppsala, Sweden) automated medium-pressure chromatography instruments with preloaded Buchi silica gel cartridges. 2,2′,3,3′,4,4′-Hexa-trimethylsilyl-α,α-D-trehalose was prepared using method from literature without any modification [29].

### 4.2. General Procedure for Esterification via Carbodiimide-Mediated Coupling

To a stirred mixture of 2,2′,3,3′,4,4′-Hexa-trimethylsilyl-α,α-D-trehalose (1 eq.; 1 mmol), aryl carboxylic acid (2.2 eq.; 2.2 mmol) and DMAP (3 eq.; 3 mmol) in anhydrous DCM (10 mL), DCC was added (3 eq.; 3 mmol) or EDC-MeI (5 eq.; 5 mmol) at 0 °C and stirred for 30 min, and then at room temperature overnight. The reaction mixture was diluted with water and then extracted with DCM. The combined organic layer was dried over MgSO_4_ and reduced in vacuo. The crude mixture was subjected to chromatography using a Biotage system with a 12 g silica column and a 0 to 20% ethyl acetate in heptane gradient. This yielded the silyl intermediate with good to excellent yields.

### 4.3. General Procedure for Deprotection of Silyl Ethers

The silyl intermediate (0.333 mmol) was dissolved in equal amounts of methylene chloride and methanol (8 mL) treated with Dowex 50WX8 resin (668.4 mg) with magnetic stirring. Upon consumption of the starting material as determined by TLC (20% methanol in methylene chloride and charring with vanillin stain) the reaction was filtered, concentrated, and chromatographed on a silica column eluting with a 40% to 80% methylene chloride to methanol gradient (Biotage system using a 12 g pre-packed column) to provide the desired product.

### 4.4. The Analytical Data for All Final Compounds

Spectral data for **3a** and **3b** were comparable to those reported in literature [18].

Synthesis of 6,6′-Bis(3,4-dibutoxybenzoyl)-α,α-D-trehalose (**2a**): diaryl ester was prepared according to general procedure for esterification and silyl deprotection to obtain 6,6′-Bis(3,4-dibutoxybenzoyl)-α,α-D-trehalose (154 mg, 72% for two steps) as white solid. ^1^H NMR (400 MHz, DMSO-*d*_6_): δ 7.56 (dd, *J* = 8.4, 1.7 Hz, 2H), 7.42 (d, *J* = 1.7 Hz, 2H), 7.06 (d, *J* = 8.4 Hz, 2H), 5.22 (d, *J* = 5.4 Hz, 2H), 4.99 (d, *J* = 4.7 Hz, 2H), 4.94 (d, *J* = 3.5 Hz, 2H), 4.92 (d, *J* = 5.9 Hz, 2H), 4.43 (d, *J* = 10.8, 2H), 4.21 (dd, *J* = 11.7, 6.6 Hz, 2H), 4.08 (t, *J* = 9.4 Hz, 2H), 4.03 (t, *J* = 6.4 Hz, 4H), 3.96 (m, 4H), 3.62 (dd, *J* = 9.0, 4.8 Hz, 2H), 3.31 (m, 2H), 3.21 (dd, *J* = 9.2, 5.4 Hz, 2H), 1.68 (m, 8H), 1.43 (m, 8H), 0.91 (m, 12H). ^13^C NMR (101 MHz, DMSO-*d*_6_): δ 165.41, 152.78, 147.91, 123.31, 121.82, 113.51, 112.44, 93.41, 72.82, 71.59, 70.37, 69.87, 68.04, 68.00, 64.06, 30.75, 30.62, 18.70, 13.67. HRMS calcd. for [C_42_H_62_O_17_ + NH_4_]^+^ requires 856.9795; found, 856.9792.

Synthesis of 6,6′-Bis(3,4-dipentoxybenzoyl)-α,α-D-trehalose (**2b**): diaryl ester was prepared according to general procedure for esterification and silyl deprotection to obtain 6,6′-Bis(3,4-dipentoxybenzoyl)-α,α-D-trehalose (135 mg, 68% for two steps) as white solid. ^1^H NMR (400 MHz, DMSO-*d*_6_): δ 7.56 (dd, *J* = 8.5, 1.8 Hz, 2H), 7.42 (d, *J* = 1.8 Hz, 2H), 7.05 (d, *J* = 8.5 Hz, 2H), 5.21 (d, *J* = 5.1 Hz, 2H), 4.99 (d, *J* = 4.1 Hz, 2H), 4.93 (dd, *J* = 7.2, 3.5 Hz, 4H), 4.43 (d, *J* = 10.3 Hz, 2H), 4.20 (dd, *J* = 11.7, 6.6 Hz, 2H), 4.08 (t, *J* = 9.3 Hz, 2H), 4.02 (t, *J* = 6.3 Hz, 4H), 3.94 (m, 4H), 3.62 (td, *J* = 8.8, 2.9 Hz, 2H), 3.31 (m, 2H), 3.21 (td, *J* = 9.1, 5.2 Hz, 2H), 1.70 (m, 8H), 1.35 (m, 16H), 0.87 (q, *J* = 7.2 Hz, 12H). ^13^C NMR (101 MHz, DMSO-*d*_6_): δ 165.40, 152.77, 147.91, 123.29, 121.80, 113.44, 112.40, 93.39, 72.80, 71.59, 70.38, 69.86, 68.28, 68.26, 28.36, 28.25, 27.75, 27.73, 21.87, 13.92. HRMS calcd. for [C_46_H_70_O_17_ + NH_4_]^+^ requires 913.0875; found: 913.0879.

Synthesis of 6,6′-Bis(3,4-dihexyloxybenzoyl)-α,α-D-trehalose (**2c**): diaryl ester was prepared according to general procedure for esterification and silyl deprotection to obtain 6,6′-Bis(3,4-dihexyloxybenzoyl)-α,α-D-trehalose (148 mg, 67% for two steps) as white solid. ^1^H NMR (400 MHz, DMSO-*d*_6_): δ ^1^H NMR (400 MHz, DMSO-d_6_) δ 7.55 (dd, *J* = 1.53, 8.50 Hz, 2H), 7.42 (d, *J* = 1.59 Hz, 2H), 7.04 (d, *J* = 8.56 Hz, 2H), 5.21 (d, *J* = 5.38 Hz, 2H), 4.99 (d, *J* = 4.77 Hz, 2H), 4.95 (d, *J* = 3.42 Hz, 2H), 4.91 (d, *J* = 5.99 Hz, 2H), 4.43 (d, *J* = 10.76 Hz, 2H), 4.20 (dd, *J* = 6.60, 11.62 Hz, 2H), 4.05–4.11 (m, 2H), 3.99–4.05 (m, 4H), 3.89–3.98 (m, 4H), 3.62 (dt, *J* = 5.01, 8.99 Hz, 2H), 3.28–3.33 (m, 2H), 3.21 (dt, *J* = 5.56, 9.20 Hz, 2H), 1.62–1.78 (m, 8H), 1.36–1.45 (m, 8H), 1.21–1.33 (m, 16H), 0.85 (q, *J* = 7.01 Hz, 12H). ^13^C NMR (101 MHz, DMSO-*d*_6_): δ 165.40, 152.79, 147.93, 123.28, 121.81, 113.44, 112.38, 93.37, 72.81, 71.61, 70.39, 69.89, 68.28, 64.07, 30.99, 30.96, 28.67, 28.54, 25.20, 25.17, 22.09, 13.83. HRMS calcd. for [C_50_H_78_O_17_ + NH_4_]^+^ requires 968.5538; found 968.6790.

Synthesis of 6,6′-Bis(3,5-dipropoxybenzoyl)-α,α-D-trehalose (**3c**): diaryl ester was prepared according to general procedure for esterification and silyl deprotection to obtain 6,6′-Bis(3,5-dipropoxybenzoyl)-α,α-D-trehalose (160.7 mg, 71% for two steps) as white solid. ^1^H NMR (400 MHz, DMSO-*d*_6_): δ 6.95–7.04 (m, 4H), 6.71 (t, *J* = 1.76 Hz, 2H), 4.93 (d, *J* = 3.13 Hz, 2H), 4.40–4.47 (m, 2H), 4.22 (dd, *J* = 6.06, 11.93 Hz, 2H), 4.02–4.09 (m, 2H), 3.76–4.00 (m, 14H), 3.60 (t, *J* = 9.19 Hz, 2H), 3.30 (dd, *J* = 3.33, 9.59 Hz, 2H), 3.21 (t, *J* = 9.59 Hz, 2H), 1.58–1.78 (m, 8H), 0.78–1.03 (m, 12H). ^13^C NMR (101 MHz, DMSO-*d*_6_): δ 165.29, 159.82, 131.63, 107.16, 106.02, 93.36, 79.78, 72.77, 71.61, 70.22, 69.70, 69.25, 64.30, 21.94, 10.33. HRMS calcd. for [C38H54O17 + NH4]^+^ requires 800.3705; found: 800.3685.

Synthesis of 6,6′-Bis(3,5-dibutoxybenzoyl)-α,α-D-trehalose (**3d**): diaryl ester was prepared according to general procedure for esterification and silyl deprotection to obtain 6,6′-Bis(3,5-dibutoxybenzoyl)-α,α-D-trehalose (201.0 mg, 72% for two steps) as white solid. ^1^H NMR (400 MHz, DMSO-*d*_6_): δ 6.91–7.12 (m, 4H), 6.62–6.81 (m, 2H), 5.23 (d, *J* = 5.48 Hz, 2H), 4.83–5.05 (m, 6H), 4.47 (d, *J* = 11.74 Hz, 2H), 4.17–4.31 (m, 2H), 3.85–4.12 (m, 10H), 3.61 (dd, *J* = 4.89, 8.80 Hz, 2H), 3.38 (s, 2H), 3.18–3.31 (m, 6H), 1.57–1.85 (m, 8H), 1.28–1.55 (m, 8H), 0.87–0.95 (m, 8H). ^13^C NMR (101 MHz, DMSO-*d*_6_): δ 165.30, 159.83, 131.62, 107.15, 72.77, 71.61, 70.25, 69.69, 67.44, 30.61, 18.66, 13.64. HRMS calcd. for [C_42_H_62_O_17_ + NH_4_]^+^ requires 856.4331; found [M + NH_4_]^+^ 856.4353.

Synthesis of 6,6′-Bis(3,5-dipentyloxybenzoyl)-α,α-D-trehalose (**3e**): diaryl ester was prepared according to general procedure for esterification and silyl deprotection to obtain 6,6′-Bis(3,5-dipentyloxybenzoyl)-α,α-D-trehalose (287.6 mg, 83% for two steps) as white solid. ^1^H NMR (400 MHz, DMSO-*d*_6_): δ 6.99–7.05 (m, 4H), 6.68–6.74 (m, 2H), 5.23 (d, *J* = 5.48 Hz, 2H), 4.92–5.00 (m, 4H), 4.88 (d, *J* = 5.87 Hz, 2H), 4.47 (d, *J* = 11.74 Hz, 2H), 4.23 (dd, *J* = 6.06, 11.54 Hz, 2H), 4.04–4.13 (m, 2H), 3.87–4.00 (m, 8H), 3.61 (dd, *J* = 4.89, 8.41 Hz, 2H), 3.13–3.28 (m, 4H), 1.59–1.74 (m, 8H), 1.22–1.43 (m, 16H), 0.87 (t, *J* = 7.04 Hz, 12H). ^13^C NMR (101 MHz, DMSO-*d*_6_): δ 165.51, 160.02, 131.82, 107.34, 106.19, 93.53, 72.96, 71.63, 70.50, 69.91, 67.93, 64.57, 28.45, 27.84, 20.05, 14.08. HRMS calcd. for [C_46_H_70_O_17_ + NH_4_]^+^ requires 912.4957; found: [M + NH_4_]^+^ 912.4981.

Synthesis of 6,6′-Bis(3,5-dihexyloxybenzoyl)-α,α-D-trehalose (**3f**): diaryl ester was prepared according to general procedure for esterification and silyl deprotection to obtain 6,6′-Bis(3,5-dihexyloxybenzoyl)-α,α-D-trehalose (201.2 mg, 75% for two steps) as white solid. ^1^H NMR (400 MHz, DMSO-*d*_6_): δ 7.02 (s, 4H), 6.71 (s, 2H), 5.23 (s, 2H), 4.97 (d, *J* = 3.91 Hz, 4H), 4.88 (br. s., 2H), 4.47 (d, *J* = 10.96 Hz, 2H), 4.17–4.28 (m, 2H), 4.08 (br. s., 2H), 3.87–4.02 (m, 8H), 3.62 (br. s., 2H), 3.13–3.31 (m, 4H), 1.60–1.79 (m, 8H), 1.17–1.47 (m, 24H), 0.72–0.98 (m, 12H). ^13^C NMR (101 MHz, DMSO-*d*_6_): δ 165.32, 165.16, 159.83, 159.67, 131.63, 131.48, 107.13, 106.97, 106.00, 105.85, 93.32, 93.16, 79.39, 74.96, 72.75, 72.60, 71.63, 70.32, 70.16, 69.55, 67.74, 67.58, 64.35, 30.96, 30.80, 28.53, 28.37, 25.13, 24.97, 22.05, 21.89, 13.88, 13.72. HRMS calcd. for [C_50_H_78_O_17_ + NH_4_]^+^ requires 968.5578; found [M + NH_4_]^+^ 968.5573.

Synthesis of 6,6′-Bis(3,5-diheptyloxybenzoyl)-α,α-D-trehalose (**3g**): diaryl ester was prepared according to general procedure for esterification and silyl deprotection to obtain **3g** as white solid (200 mg, 88% for two steps). ^1^H NMR (400 MHz, DMSO-*d*_6_): δ 7.02 (s, 4H), 6.70 (d, *J* = 2.6 Hz, 2H), 5.24 (d, *J* = 5.2 Hz, 2H), 5.04–4.86 (m, 6H), 4.47 (d, *J* = 11.3 Hz, 2H), 4.22 (dd, *J* = 11.8, 6.4 Hz, 2H), 4.07 (t, *J* = 8.3 Hz, 2H), 3.96 (d, *J* = 6.8 Hz, 8H), 3.62 (td, *J* = 9.0, 4.4 Hz, 2H), 3.21 (td, *J* = 9.3, 5.3 Hz, 2H), 1.67 (p, *J* = 6.8 Hz, 8H), 1.45–1.14 (m, 34H), 0.84 (t, *J* = 6.5 Hz, 12H). ^13^C NMR (101 MHz, DMSO-*d*_6_): δ 165.78, 160.28, 132.10, 107.58, 106.46, 93.76, 73.23, 72.10, 70.79, 70.17, 68.18, 64.85, 40.60, 40.39, 40.19, 39.98, 39.77, 39.56, 39.35, 31.69, 29.04, 28.89, 25.89, 22.52, 14.38. HRMS calcd. for [C_54_H_86_O_17_ + NH_4_]^+^ requires 1024.6163; found 1024.6011.

Synthesis of 6,6′-Bis(3,5-dioctyloxybenzoyl)-α,α-D-trehalose (**3h**): diaryl ester was prepared according to general procedure for esterification and silyl deprotection to obtain 6,6′-Bis(3,5-dioctyloxybenzoyl)-α,α-D-trehalose (155.2 mg, 71% for two steps) as white solid. ^1^H NMR (400 MHz, DMSO-*d*_6_): δ 6.83 (tdd, *J* = 1.66, 3.57, 5.23 Hz, 4H), 6.51 (d, *J* = 4.30 Hz, 2H), 4.96–5.08 (m, 2H), 4.74–4.80 (m, 4H), 4.68 (ddd, *J* = 1.96, 4.60, 8.31 Hz, 2H), 4.26 (d, *J* = 7.83 Hz, 2H), 4.02 (d, *J* = 6.26 Hz, 2H), 3.88 (d, *J* = 6.26 Hz, 2H), 3.75 (br. s., 8H), 3.43 (br. s., 2H), 2.93–3.11 (m, 4H), 1.47 (br. s., 8H), 0.93–1.24 (m, 40H), 0.64 (ddd, *J* = 1.96, 3.52, 5.09 Hz, 12H). ^13^C NMR (101 MHz, DMSO-*d*_6_): δ 165.30, 159.80, 131.64, 107.11, 79.58, 75.09, 72.76, 71.63, 70.3, 69.70, 67.71, 64.37, 31.24, 28.71, 28.66, 28.55, 25.46, 22.08, 13.92. HRMS calcd. for [C_54_H_94_O_17_ + NH_4_]^+^ requires 1080.6828; found 1080.6821.

Synthesis of 6,6′-Bis(3,5-didecyloxybenzoyl)-α,α-D-trehalose (**3i**): diaryl ester was prepared according to general procedure for esterification and silyl deprotection to obtain 6,6′-Bis(3,5-didecyloxybenzoyl)-α,α-D-trehalose (141.7 mg, 77% for two steps) as white solid. ^1^H NMR (400 MHz, DMSO-*d*_6_): δ 7.02 (s, 4H), 6.66 (s, 2H), 5.20 (d, *J* = 5.4 Hz, 2H), 4.97(d, *J* = 3.9 Hz, 4H), 4.86 (d, *J* = 5.9 Hz, 2H), 4.46 (d, *J* = 11.5 Hz, 2H), 4.21 (dd, *J* = 11.5, 6.3 Hz, 2H), 4.07 (t, *J* = 7.6 Hz, 2H), 3.93 (br, 8H), 3.63 (m, 2H), 3.30 (m, 2H), 3.20 (br, 2H), 1.65 (m, 8H), 1.35 (br, 8H), 1.19 (br, 48H), 0.82 (t, *J* = 6.4 Hz, 12 H). ^13^C NMR (101 MHz, DMSO-*d*_6_): δ 165.28, 159.77, 131.65, 128.39, 107.09, 105.98, 93.17, 79.55, 72.78, 71.63, 70.37, 69.69, 67.66, 64.39, 31.32, 29.01, 28.97, 28.73, 28.54, 25.44, 22.11, 13.89. HRMS calcd. for [C_66_H_110_O_17_ + NH_4_]^+^ requires 1192.8082; found 1192.8099.

Synthesis of 6,6′-Bis(3,4,5-triethoxybenzoyl)-α,α-D-trehalose (**4a**): diaryl ester was prepared according to general procedure for esterification and silyl deprotection to obtain 6,6′-Bis(3,4,5-triethoxybenzoyl)-α,α-D-trehalose (74.8 mg, 52% for two steps) as white solid. ^1^H NMR (400 MHz, DMSO-*d*_6_): δ 7.06 (s, 4H), 5.11 (d, *J* = 5.01 Hz, 2H), 4.87 (d, *J* = 4.77 Hz, 2H), 4.79–4.85 (m, 4H), 4.32 (d, *J* = 11.37 Hz, 2H), 4.03–4.14 (m, 2H), 3.80–3.99 (m, 14H), 3.43–3.54 (m, 2H), 3.15 (d, *J* = 3.67 Hz, 2H), 3.01–3.10 (m, 2H), 1.19 (t, *J* = 6.79 Hz, 12H), 1.10 (t, *J* = 6.97 Hz, 6H). ^13^C NMR (101 MHz, DMSO-*d*_6_): δ 165.42, 152.45, 141.44, 124.72, 107.57, 93.44, 72.97, 71.89, 70.68, 68.30, 64.29, 15.83, 14.83. HRMS calcd. for [C_38_H_54_O_19_ + NH_4_]^+^ requires 832.3603; found: 832.3618.

Synthesis of 6,6′-Bis(3,4,5-tripropoxybenzoyl)-α,α-D-trehalose (**4b**): diaryl ester was prepared according to general procedure for esterification and silyl deprotection to obtain 6,6′-Bis(3,4,5-tripropoxybenzoyl)-α,α-D-trehalose (184.2 mg, 78% for two steps) as white solid. ^1^H NMR (400 MHz, DMSO-*d*_6_): δ 7.19 (s, 4H), 5.24(d, *J* = 5.2 Hz, 2H), 4.99 (dd, *J* = 8.4, 4.5 Hz, 4H), 4.92 (d, *J* = 6.0 Hz, 2H), 4.47 (d, *J* = 11.0 Hz, 2H), 4.17 (dd, *J* = 11.0, 4.6 Hz, 2H), 4.08 (t, *J* = 8.5 Hz, 2H), 3.92 (m, 12H), 3.62 (m, 2H), 3.29 (m, 2H), 3.18 (m, 2H), 1.72 (m, 8H), 1.64 (m, 4H), 0.97 (t, *J* = 7.3 Hz, 18H). ^13^C NMR (101 MHz, DMSO-*d*_6_): δ 165.02, 152.11, 141.23, 124.23, 107.06, 92.93, 79.32, 74.00, 72.76, 72.54, 70.45, 70.28, 69.66, 22.81, 21.94, 10.22, 10.20. HRMS calcd. for [C_44_H_66_O_19_ + NH_4_]^+^ requires 916.4552; found 916.4562.

Synthesis of 6,6′-Bis(3,4,5-tributoxybenzoyl)-α,α-D-trehalose (**4c**): diaryl ester was prepared according to general procedure for esterification and silyl deprotection to obtain 6,6′-Bis(3,4,5-tributoxybenzoyl)-α,α-D-trehalose (202.21 mg, 85% for two steps) as white solid. ^1^H NMR (400 MHz, DMSO-*d*_6_): δ 7.19 (s, 4H), 5.24 (d, *J* = 4.7 Hz, 2H), 4.99 (m, 4H), 4.91 (d, *J* = 5.8 Hz, 2H), 4.48 (d, *J* = 9.9 Hz, 2H), 4.16 (m, 2H), 4.07 (t, *J* = 7.6 Hz, 2H), 3.95 (m, 12H), 3.64 (br, 2H), 3.29 (br, 2H), 3.17 (m, 2H), 1.68 (m, 8H), 1.60 (m, 4H), 1.44 (m, 12H), 0.89 (t, *J* = 7.5 Hz, 18H). ^13^C NMR (101 MHz, DMSO-*d*_6_): δ 165.23, 152.37, 141.44, 124.48, 107.23, 93.06, 79.55, 72.74, 72.15, 71.69, 70.56, 69.80, 68.01, 64.55, 31.75, 30.81, 18.72, 18.61, 13.61. HRMS calcd. for [C_50_H_78_O_19_ + NH_4_]^+^ requires 1000.5476; found 1000.5472.

Synthesis of 6,6′-Bis(3,4,5-tripentyloxybenzoyl)-α,α-D-trehalose (**4d**): diaryl ester was prepared according to general procedure for esterification and silyl deprotection to obtain 6,6′-Bis(3,4,5-tripentyloxybenzoyl)-α,α-D-trehalose (181 mg, 76% for two steps) as white solid. ^1^H NMR (400 MHz, DMSO-*d*_6_): δ 7.18 (s, 4H), 5.25 (d, *J* = 5.8 Hz, 2H), 5.00 (dd, *J* = 12.3, 4.2 Hz, 4H), 4.93 (d, *J* = 5.9 Hz, 2H), 4.49 (d, *J* = 10.4 Hz, 2H), 4.16 (dd, *J* = 11.3, 7.5 Hz, 2H), 4.08(t, *J* = 9.0 Hz, 2H), 3.94 (m, 12H), 3.63 (dt, *J* = 9.1, 3.4 Hz, 2H), 3.30 (m, 2H), 3.16 (m, 2H), 1.69 (m, 8H), 1.63 (m, 4H), 1.39 (m, 12H), 1.31 (m, 12H), 0.86 (m, 18H). ^13^C NMR (101 MHz, DMSO-*d*_6_): δ 165.23, 152.34, 14.37, 124.46, 107.16, 93.04, 72.72, 72.48, 71.68, 70.60, 69.78, 68.28, 29.38, 28.44, 27.74, 27.69, 21.88, 21.84, 13.87. HRMS calcd. for [C_56_H_90_O_19_ + NH_4_]^+^ 1084.6375; found 1084.7693.

Synthesis of 6,6′-Bis(3,4,5-trihexyloxybenzoyl)-α,α-D-trehalose (**4e**): diaryl ester was prepared according to general procedure for esterification and silyl deprotection to obtain 6,6′-Bis(3,4,5-trihexyloxybenzoyl)-α,α-D-trehalose (205.8 mg, 82% for two steps) as white solid. ^1^H NMR (400 MHz, DMSO-*d*_6_): δ 7.18 (s, 4H), 5.24 (d, *J* = 4.8 Hz, 2H), 4.98 (d, *J* = 3.7 Hz, 4H), 4.91 (d, *J* = 6.3 Hz, 2H), 4.48 (d, *J* = 11.0 Hz, 2H), 4.15 (m, 2H), 4.07 (t, *J* = 7.9 Hz, 2H), 3.93 (m, 12H), 3.63 (m, 2H), 3.29 (br, 2H), 3.15 (m, 2H), 1.67 (m, 12H), 1.48 (m, 12H), 1.44 (m, 24H), 0.88 (t, *J* = 7.5 Hz, 18H). ^13^C NMR (101 MHz, DMSO-*d*_6_): δ 165.22, 152.33, 141.36, 124.47, 107.13, 92.98, 79.66, 72.72, 72.49, 71.69, 70.62, 69.77, 68.27, 64.59, 31.08, 30.96, 29.71, 28.73, 25.24, 25.17, 22.12, 22.08, 13.84, 13.80. HRMS calcd. for [C_62_H_102_O_19_ + NH_4_]^+^ requires 1168.7354; found 1168.7345.

Synthesis of 6,6′-Bis(3,4,5-triheptyloxybenzoyl)-α,α-D-trehalose (**4f**): diaryl ester was prepared according to general procedure for esterification and silyl deprotection to obtain 6,6′-Bis(3,4,5-triheptyloxybenzoyl)-α,α-D-trehalose (181 mg, 76% for two steps) as white solid. ^1^H NMR (400 MHz, DMSO-*d*_6_): δ 7.17 (s, 4H), 5.23 (d, *J* = 5.4 Hz, 2H), 4.99 (d, *J* = 4.2 Hz, 4H), 4.91 (d, *J* = 5.9 Hz, 2H), 4.48 (d, *J* = 10.4 Hz, 2H), 4.15 (dd, *J* = 7.5, 11.2 Hz, 2H), 4.07 (t, *J* = 9.2 Hz, 2H), 3.91 (m, 12H), 3.63 (m, 2H), 3.29 (m, 2H), 3.16 (m, 2H), 1.63 (m, 12H), 1.38 (m, 12H), 1.22 (br, 36H), 0.82 (m, 18H). ^13^C NMR (101 MHz, DMSO-*d*_6_): δ 165.21, 152.32, 141.36, 124.48, 107.12, 92.96, 72.72, 72.46, 71.68, 70.62, 69.74, 68.23, 64.55, 31.37, 31.29, 29.79, 28.78, 28.58, 28.46, 25.55, 25.49, 22.09, 22.04, 13.86. HRMS calcd. for [C_68_H_114_O_19_ + NH_4_]^+^ requires 1253.6795; found 1253.6787.

Synthesis of 6,6′-Bis(3,4,5-trioctyloxybenzoyl)-α,α-D-trehalose (**4g**): diaryl ester was prepared according to the general procedure for esterification and silyl deprotection to obtain 6,6′-Bis(3,4,5-trioctyloxybenzoyl)-α,α-D-trehalose (215.3 mg, 84% for two steps) as a white solid. ^1^H NMR (400 MHz, DMSO-*d*_6_): δ 7.13 (s, 4H), 5.17 (br, 2H), 4.98 (d, *J* = 8.9 Hz, 4H), 4.85 (d, *J* = 4.4 Hz, 2H), 4.46 (d, *J* = 9.2 Hz, 2H), 4.14 (br, 2H), 4.04 (br, 2H), 3.84 (br, 12H), 3.64 (br, 2H), 3.28 (br, 2H), 3.16 (br, 2H), 1.60 (br, 12H), 1.33 (br, 12H), 1.16 (br, 48H), 0.76 (br, 18H). ^13^C NMR (101 MHz, DMSO-*d*_6_): δ 165.12, 152.23, 141.41, 124.47, 107.10, 92.91, 72.78, 71.78, 71.63, 70.62, 69.70, 68.14, 64.42, 31.34, 31.26, 29.66, 28.95, 28.77, 28.76, 28.73, 25.59, 25.52, 22.08, 13.79, 13.76. HRMS calcd. for [C_74_H_126_O_19_ + NH_4_]^+^ requires 1336.9232; found 1336.9222.

Synthesis of 6,6′-Bis(3,4,5-tridecyloxybenzoyl)-α,α-D-trehalose (**4h**): diaryl ester was prepared according to general procedure for esterification and silyl deprotection to obtain 6,6′-Bis(3,4,5-tridecyloxybenzoyl)-α,α-D-trehalose trehalose (154 mg, 72% for two steps) as white solid. ^1^H NMR (400 MHz, Pyridine-*d_5_*): δ 7.75 (d, *J* = 3.13 Hz, 4H), 6.06 (d, *J* = 3.13 Hz, 2H), 5.30–5.42 (m, 4H), 5.00 (dd, *J* = 7.24, 10.76 Hz, 2H), 4.90 (br. s., 2H), 4.81 (t, *J* = 9.00 Hz, 2H), 4.34 (d, *J* = 9.78 Hz, 2H), 4.28 (t, *J* = 6.26 Hz, 4H), 4.17 (t, *J* = 9.19 Hz, 2H), 4.07 (br. s., 12H), 1.16–2.03 (m, 96H), 0.83–0.98 (m, 18H). ^13^C NMR (101 MHz, Pyridine-*d_5_*): δ 166.36, 153.19, 153.14, 142.54, 142.50, 125.56, 125.51, 108.23, 108.19, 95.38, 95.3, 74.79, 74.74, 73.36, 73.35, 73.31, 72.45, 72.41, 71.47, 71.42, 68.95, 68.90, 65.78, 65.77, 65.75, 65.73, 65.72, 31.93, 31.88, 30.66, 30.61, 29.84, 29.82, 29.79, 29.73, 29.69, 29.66, 29.64, 29.61, 29.53, 29.51, 29.49, 29.46, 29.44, 29.41, 29.69, 29.36, 26.31, 26.26, 26.22, 22.73, 22.68, 14.05, 14.00, 13.99. HRMS calcd. for [C_86_H_150_O_19_ + NH_4_]^+^ requires 1505.1135; found 1505.1132.

### 4.5. Isolation of Human PBMCs

Peripheral blood mononuclear cells were isolated from blood samples obtained from healthy adult donors, following approval by University of Montana Institutional Review Board (IRB protocol no. 43-16) and obtaining informed consent from each donor. PBMCs were isolated as described previously [18].

### 4.6. PBMCs Stimulation and Cytokine Analysis

Compounds were dissolved in 200 proof ethanol (Decon Labratories, King of Prussia, PA, USA) and stored for 24 h at room temperature. Prior to plate coating, compounds were heated to 40 °C for 2 min in sonicating water bath and then serially diluted to desired concentrations in 96-well tissue culture plate and allowed to air-dry overnight at room temperature. Total of 5.0 × 10^5^ freshly isolated PBMCs were added to each well in RPMI-1640 (Thermo Fisher Scientific, Waltham, MA, USA) containing 5% autologous plasma and cultured for 24 h at 37 °C. Cell supernatants were harvested by centrifugation (500× *g*, 5 min, RT) and frozen at −20 °C until time of analysis. Secreted IL-6 was measured in supernatants using DuoSet Human IL-6 ELISA (R&D Systems; Minneapolis, MN, USA) as per manufacturer’s instructions (Figure 3). Additional cytokine analysis in supernatants was performed using MSD U-PLEX Assay as (Meso Scale Discovery; Rockville, MD, USA) per manufacturer’s instructions.

### 4.7. Statistical Analysis

Experiments with hPBMCs were performed with cells from three donors. All data were analyzed using GraphPad Prism 10 software (Graph Pad, Boston, MA, USA). Significant differences between compounds were analyzed using 2-way ANOVA with Tukey’s multiple comparisons test. Significances were considered as follows: ns = *p* > 0.05, * *p* ≤ 0.05, ** *p* ≤ 0.01, *** *p* ≤ 0.001, **** *p* ≤ 0.0001.

### 4.8. Molecular Docking Methods

A molecular docking study using the Cresset Flare software suite (Version 8) (License; SPK-university of montana-2023-10-25), was conducted to confirm and analyze the impact of the substitution pattern on the binding characteristics of the C_5_-lipidated derivatives (2b, 3e, and 4d), within the binding site of the human Macrophage-Inducible C-type Lectin receptor (hMincle). The X-ray protein structure of hMincle (PDB ID: 3WH2) was obtained from the Protein Data Bank (PDB), https://www.rcsb.org/ (accessed on 15 June 2024); the protein was prepared for this docking study using the default protocol for protein preparation in Flare, then the water molecules were removed. The co-crystalized citrate was used to define the active site for docking near the Ca^+2^ co-ordination site. The method employed in this molecular modeling study leveraged the structural data from bovine Mincle (PDB: 4KZV), which has a well-characterized carbohydrate recognition domain and has been co-crystallized with trehalose. Due to the absence of a crystal structure for human Mincle (hMincle) in complex with trehalose, we utilized a docking approach to predict the interaction of trehalose with hMincle. This docking simulation aimed to generate a model of hMincle with trehalose bound to it in a manner that mimics the bovine Mincle–trehalose complex. Subsequently, the investigated compounds of interest were docked into the active site of hMincle using the docked trehalose as the template ligand. To validate our docking setup, we redocked trehalose into the C-type carbohydrate recognition domain (CRD) of human Mincle (PDB ID: 3WH2). As shown in Figure 5B, this redocking successfully replicated the binding pattern of the co-crystallized trehalose within the CRD of bovine Mincle (PDB ID: 4KZV). Specifically, the simulation confirmed that the first glucose residue of trehalose chelates with Ca^2+^ in the CRD, similar to the co-crystallized citrate in human Mincle and trehalose in bovine Mincle. Additionally, the docking pose reproduced all the significant interactions established by the co-crystallized ligands with key amino acids in both the primary and secondary binding sites.

The compounds were prepared with meticulous attention to preserving the structural integrity of the trehalose moiety and maintaining its correct stereochemistry throughout the process. Initially, the trehalose structure was obtained in SMILES format from PubChem, ensuring the correct representation of its stereochemical configuration. Using this trehalose SMILES as a template, the structures of the compounds of interest were constructed and optimized. To prevent issues, such as flipping of the trehalose moiety and unintended changes in stereochemistry during the normal energy minimization step, a conformational hunt and aligns were performed using Flare software (Version 8). This approach ensured that the compounds maintained realistic conformations and adhered to the intended stereochemistry derived from the trehalose template. Subsequently, we employed a flexible docking protocol allowing for the rotation of ester bonds within the docked compounds and flexible H-bond donors in the protein. This flexibility accounted for the variations in the orientation of the functional groups and enhanced the accuracy of our predictions. This flexibility in the docking process accommodated potential variations in the orientation of the functional groups, thereby enhancing the accuracy of predicting how the compounds interacted with the target protein. Furthermore, the docked poses of all the investigated compounds (**2b**, **3e**, and **4d**) showed that the trehalose moiety was perfectly aligned with the reference docked trehalose (refer to Figure 6B). This alignment was critical for validating the reliability of the docking poses and correlating them with the experimental biological activity.

### 4.9. Molecular Dynamic Simulation Methods

The molecular dynamics (MDs) simulations were performed using the latest MDs simulation platform provided by Cresset, with the starting configuration derived from the flexible docking pose of compound **4d** in complex with hMincle. The simulation was carried out for 100 nanoseconds (ns) using an explicit solvation model with TIP3P water molecules. The simulation box was geometrically defined as a truncated octahedron to optimize the spatial distribution of the solvent and minimize the edge effects, and GCNCMC (Generalized Constrained Normal Coordinate Monte Carlo) was employed to enhance the equilibration of the water molecules. The system was maintained at a constant temperature of 298 Kelvin (K) and a pressure of 1 bar, with the ionic strength of the solvent neutralized to prevent electrostatic artifacts. Open Force Field (OpenFF) (version 2.0.0) was used to model the interactions between atoms. An analysis of the resulting trajectories was conducted using the Flare Dynamics module, which provided advanced tools for evaluating and interpreting the dynamic behavior of the complex.

## 5. Conclusions

The substitution and lipidation effects of aryl trehalose derivatives were evaluated in a new library of small-molecule Mincle agonist candidates. The compounds were easily prepared from readily available starting materials and in a high yield. The lead compound **4d** induced high levels of Th17-polarizing cytokines. The substitution evaluation highlighted the trend of increased cytokine response from the tri-substituted derivatives, owing to the tri-alkoxy functional groups in close contact with the Mincle CRD. The optimal lipidation length for hPBMCs’ cytokine response appears to be the middle-to-long carbon chain length, C_5_ to C_8_. An in silico evaluation also correlated with the in vitro findings of substitution importance, as **4d** exhibited dual chelation with the Ca^2+^ ion and the highest predicted affinity with the human Mincle CRD compared to the di-substituted C_5_ derivatives **2b** and **3e**. Cytokine profiling revealed that IL-6 is a useful tool for differentiating the compounds’ potency, but extending the analysis to other Th-effector and -polarizing cytokines was helpful in discerning the SAR.

The work presented here underscores the importance of SAR studies to advance the development of vaccine adjuvant candidates. The further development of the compounds in this family will focus on a biocompatible delivery platform suitable for co-delivery and the presentation of the Mincle agonist in tandem with a target antigen [24,30]. The biological activity of active pharmaceutical ingredients is highly dependent on the compound distribution and delivery to the target cell upon administration. In the context of targeting extracellular CLRs, such as Mincle, the formulation and presentation of Mincle agonists play a crucial role, substantially influencing their distribution and activity both in vitro and in vivo. Furthermore, vaccine adjuvants that efficiently co-deliver with an antigen should be also considered. As all the compounds used in this study were examined through plate deposition that cannot be translated to in vivo, a safe and biocompatible nanoparticle formulation suitable for in vivo co-delivery and presentation of the Mincle agonist along with the antigen have recently been evaluated [24,30].

## 6. Patents

Burkhart, D.; Evans, J.; Ettenger, G.; Ryter, K.; Smith, A. Diaryl trehalose compounds and uses thereof. World Intellectual Property Office Patent no. WO2019169313A1.

## Figures and Tables

**Table 1:** Focused library of 6,6′-diaryl trehalose compounds.

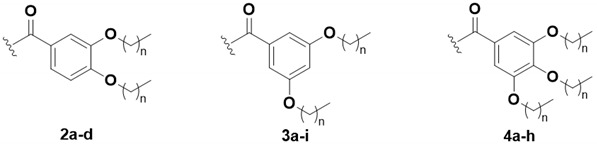		
**Compound**	**n**	**Coupling and Deprotection** **Yield [%]**
**2a**	3	72
**2b**	4	68
**2c**	5	67
**2d**	6	45
**3a**	0	42 ^1^
**3b**	1	49 ^1^
**3c**	2	71
**3d**	3	72
**3e**	4	83
**3f**	5	75
**3g**	6	88
**3h**	7	71
**3i**	9	77
**4a**	1	52
**4b**	2	75
**4c**	3	85
**4d**	4	76
**4e**	5	82
**4f**	6	76
**4g**	7	84
**4h**	9	72

^1^ Compound previously reported in [18].

## Data Availability

The original chemical contributions presented in this study are included in the article and supplementary material; further data and analysis inquiries can be directed to the corresponding author.

## Supplementary Materials

The following supporting information can be downloaded at: https://www.mdpi.com/article/10.3390/ijms251810031/s1.
