# Supplementary material for: Systematic Evaluation of Regiochemistry and Lipidation of Aryl Trehalose Mincle Agonists
Source: Int J Mol Sci. 2024 Sep 18;25(18):10031. doi: 10.3390/ijms251810031 (PMC11432005; doi:10.3390/ijms251810031)
Supplement: Supplementary file 1 [file ijms-25-10031-s001.zip › ijms-3174621-supplementary.pdf]

Systematic Evaluation of Regiochemistry and Lipidation of Aryl Trehalose Mincle Agonists

Asia Marie S. Riel[1], Viktoria Rungelrath[2], Tamer Elwaie[1,4], Omer K. Rasheed[1,3], Linda Hicks[2], George Ettenger[1], Dai-Chi You[1], Mira Smith[2], Cassandra Buhl[2,3], Walid Abdelwahab[2], Shannon M. Miller[2,3], Alyson J. Smith[2,3], David Burkhart[2,3], Jay T. Evans[2,3] and Kendal T. Ryter [1,3,*]

[1] Department of Chemistry and Biochemistry, Center for Translational Medicine, University of Montana, Missoula, MT 59812, USA; asiamarie.riel@mso.umt.edu (A.M.S.R.); tamer.elwaie@mso.umt.edu (T.A.E.); georgeettenger@gmail.com (G.E.); dai-chi.you@mso.umt.edu (D.-C.Y.)

[2] Department of Biomedical and Pharmaceutical Sciences, Center for Translational Medicine, University of Montana, Missoula, MT 59812, USAviktoria.rungelrath@mso.umt.edu (V.R.); linda.hicks@mso.umt.edu (L.H.); miradevismith@gmail.com (M.S.); cassie.buhl@gmail.com (C.B.); walid.abdelwahab@mso.umt.edu (W.A.); shannon.m.miller@inimmune.com (S.M.M.); smith.alysonj@gmail.com (A.J.S.); david.burkhart@mso.umt.edu (D.B.); jay.evans@mso.umt.edu (J.T.E.)

[3] Department of Pharmaceutical Chemistry, Faculty of Pharmacy, Cairo University, Cairo 11562, Egypt

[4] Inimmune Corporation, Missoula, MT 59802, USA

[*] Correspondence: kendal.ryter@mso.umt.edu

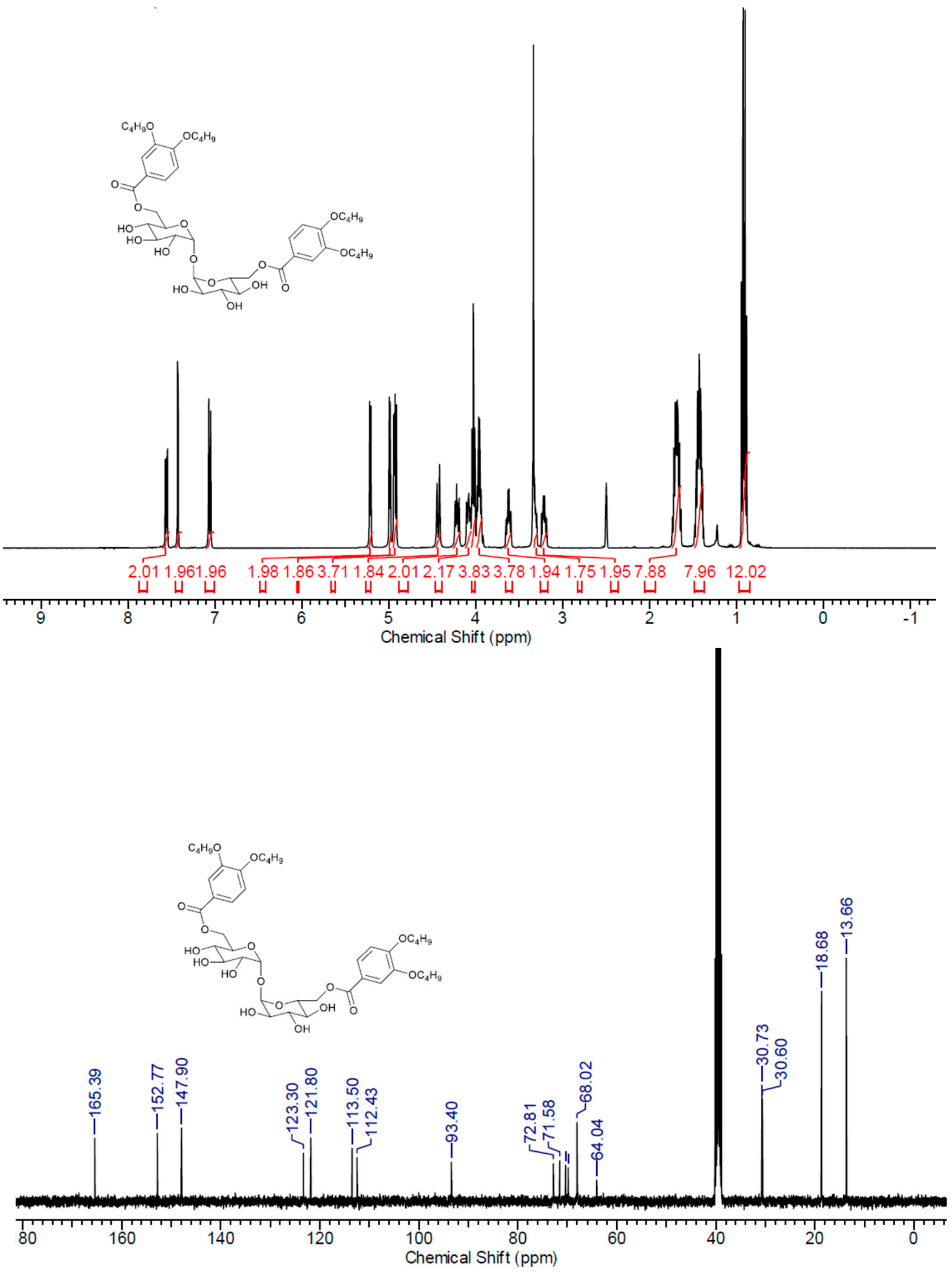

Figure S1: 6,6′-Bis(3,4-dibutoxybenzoyl)-α,α-D-trehalose (**2a**): ^{1}H NMR (400 MHz, DMSO-D$_6$) and ^{13}C NMR (101 MHz, DMSO-D$_6$).

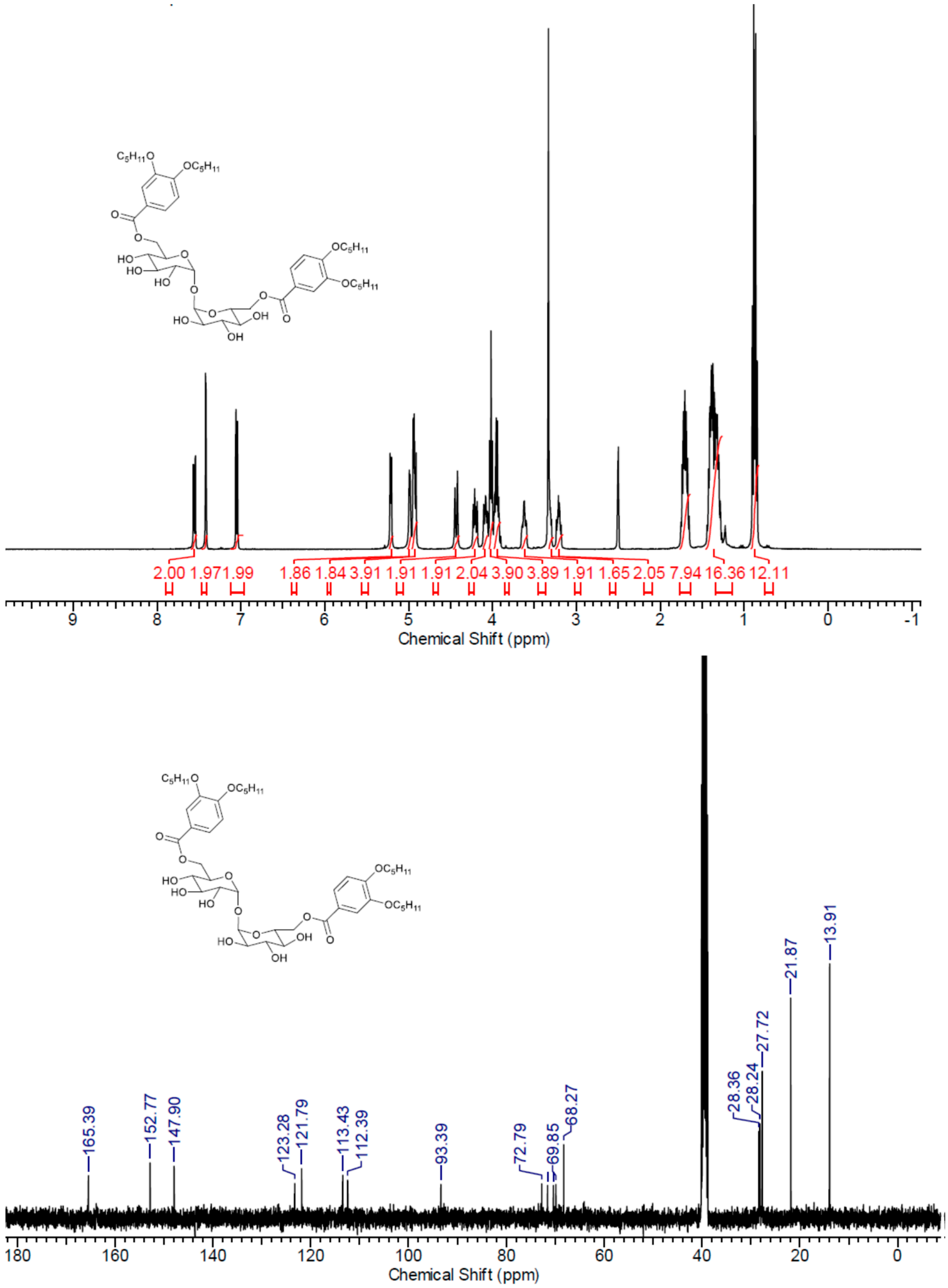

Figure S2: 6,6´-Bis(3,4-dipentoxybenzoyl)-α,α-D-trehalose (**2b**): ^{1}H NMR (400 MHz, DMSO-D_6) and ^{13}C NMR (101 MHz, DMSO-D_6).

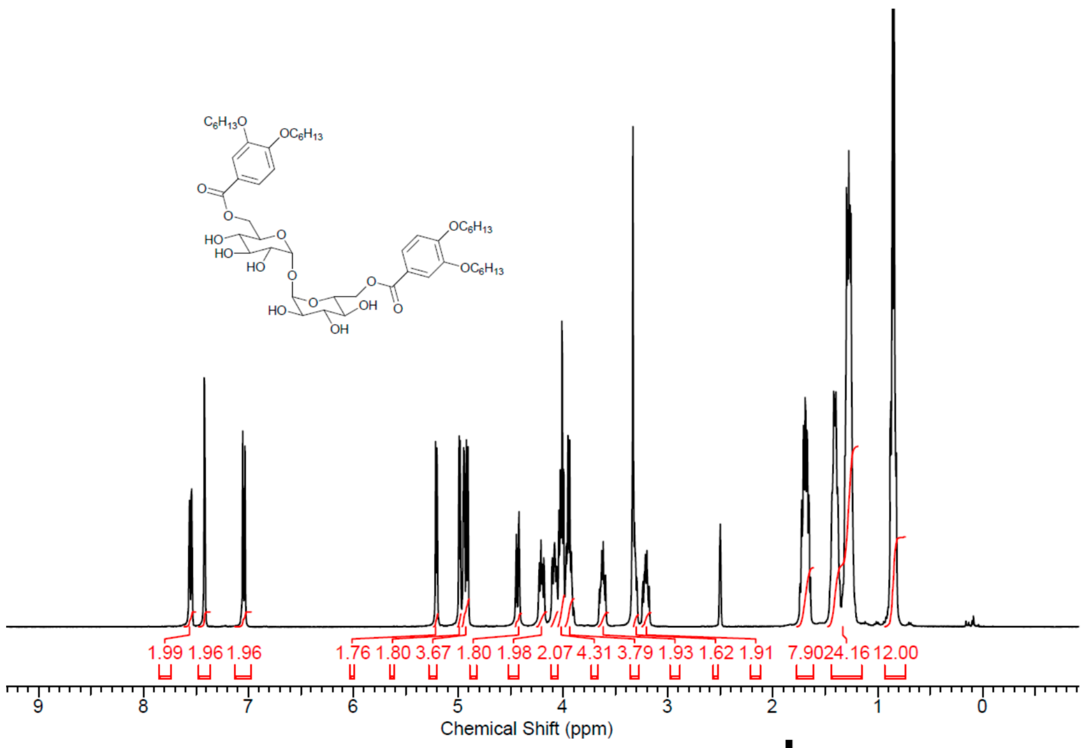

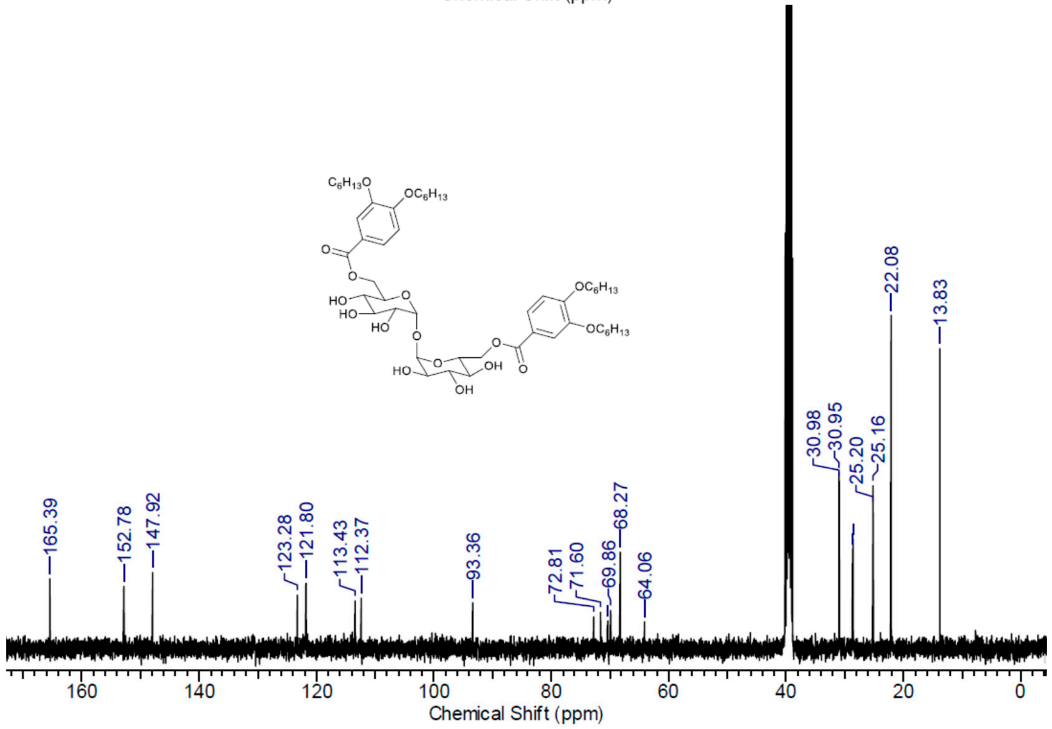

Figure S3: 6,6´-Bis(3,4-dihexyloxybenzoyl)-α,α-D-trehalose (**2c**): ^{1}H NMR (400 MHz, DMSO-D_6) and ^{13}C NMR (101 MHz, DMSO-D_6).

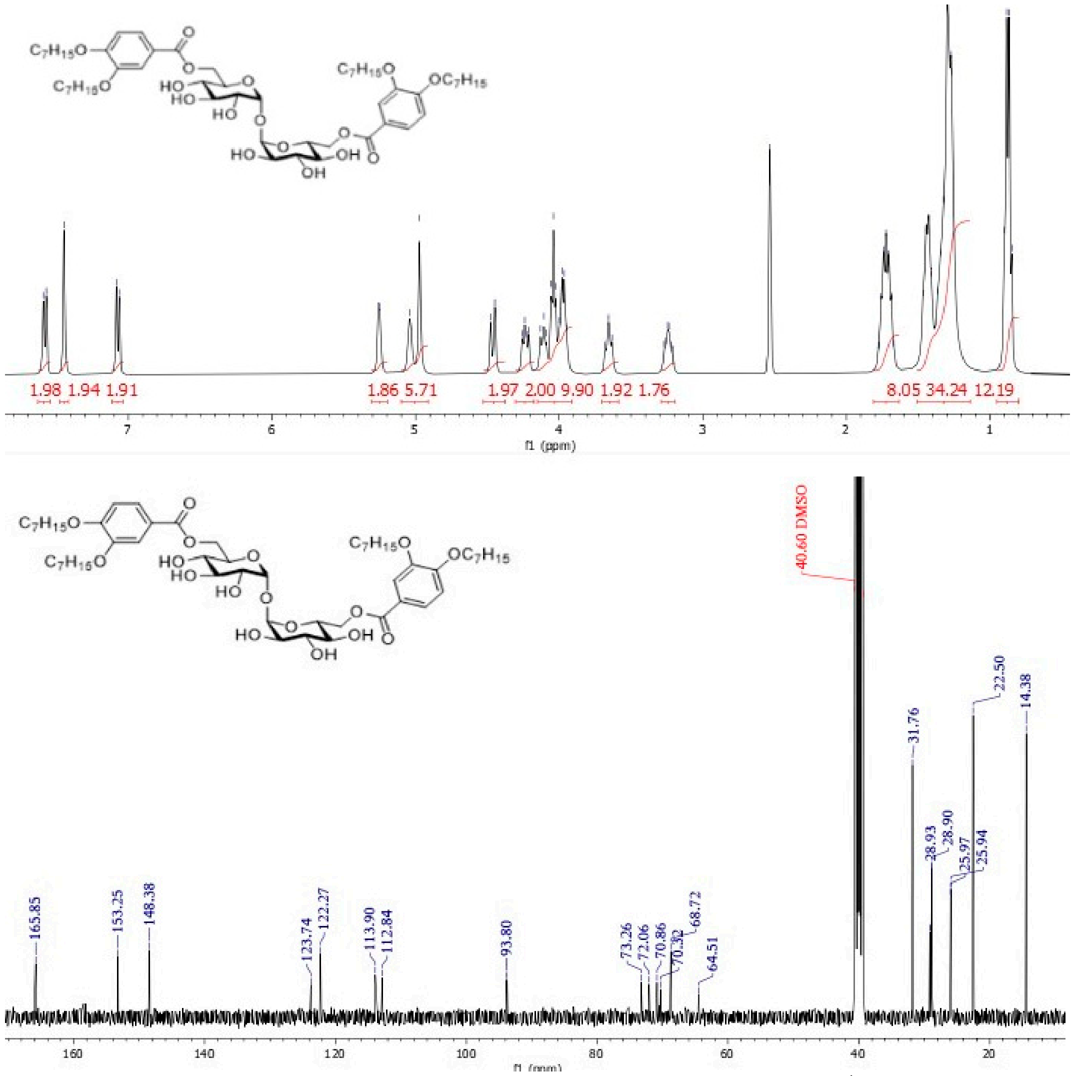

Figure S4: 6,6´-Bis(3,4-diheptyloxybenzoyl)-α,α-D-trehalose (**2d**): ^{1}H NMR (400 MHz, DMSO-D_6) and ^{13}C NMR (101 MHz, DMSO-D_6).

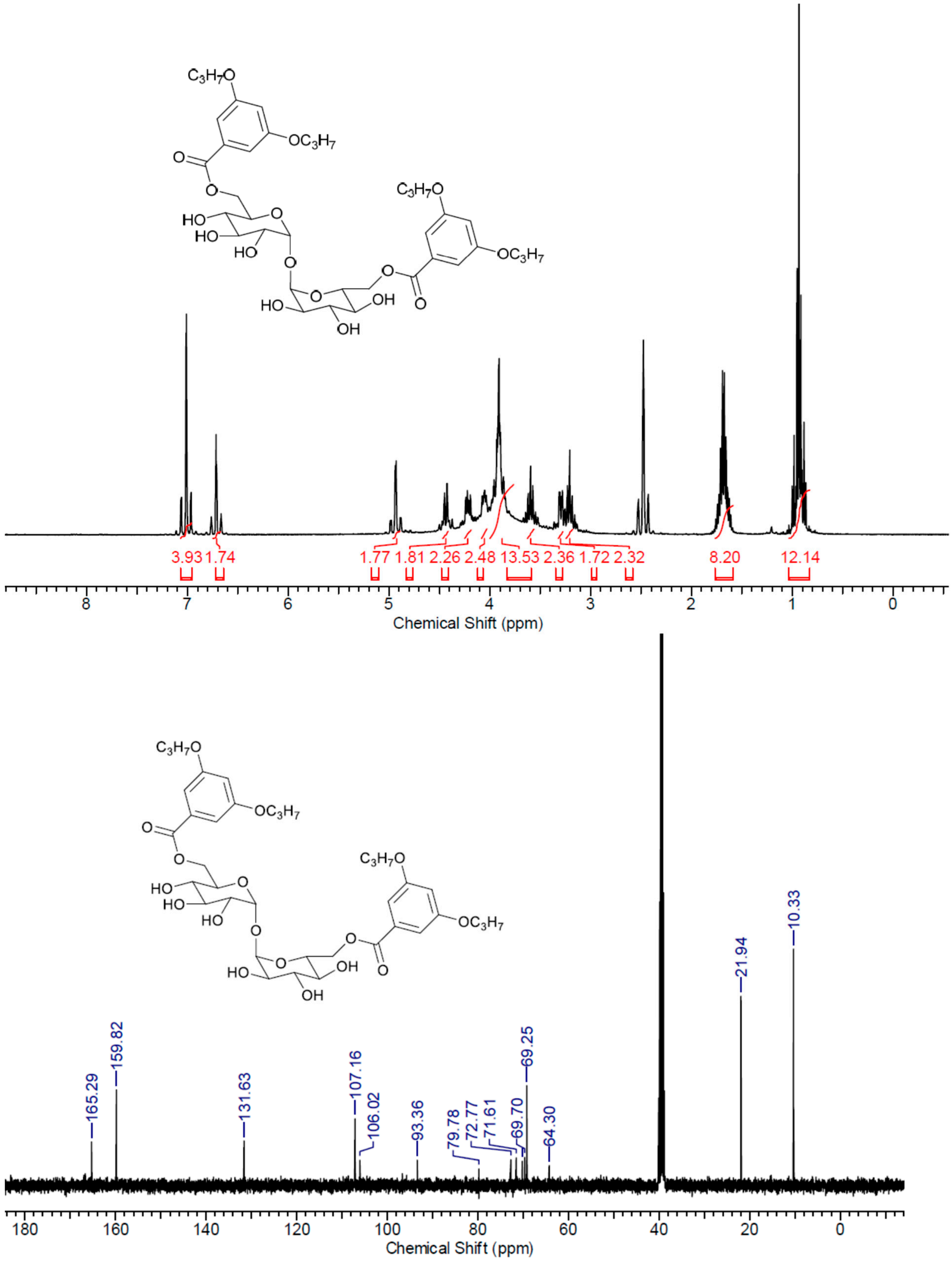

Figure S5: 6,6′-Bis(3,5-dipropoxybenzoyl)-α,α-D-trehalose (**3c**): ^{1}H NMR (400 MHz, DMSO-D_6) and ^{13}C NMR (101 MHz, DMSO-D_6).

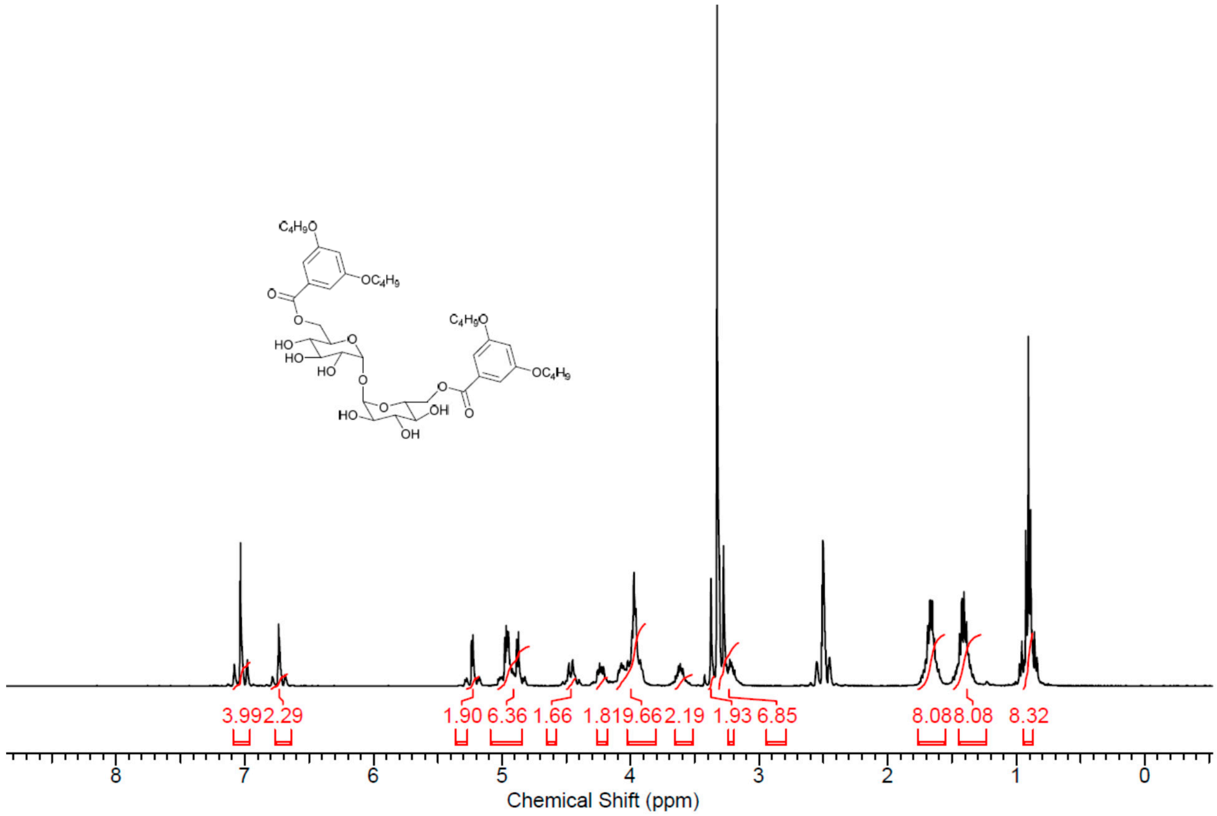

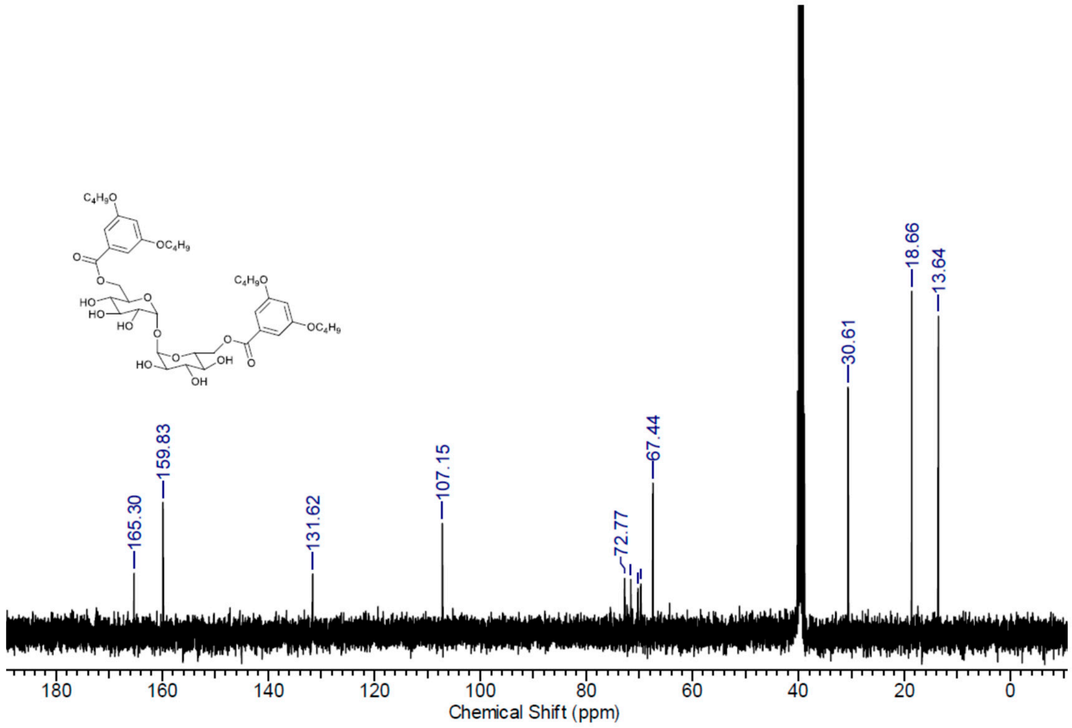

Figure S6: 6,6´-Bis(3,5-dibutoxybenzoyl)-α,α-D-trehalose (**3d**): ^{1}H NMR (400 MHz, DMSO-D$_6$) and ^{13}C NMR (101 MHz, DMSO-D$_6$).

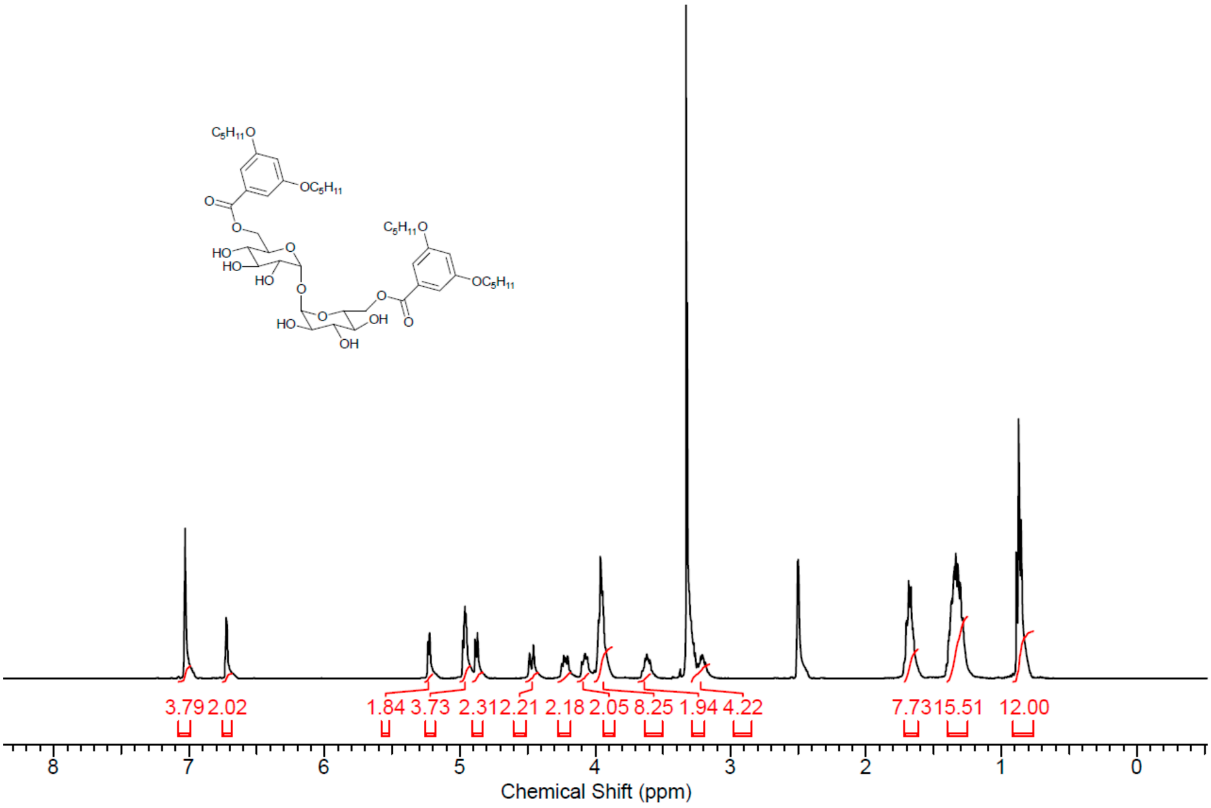

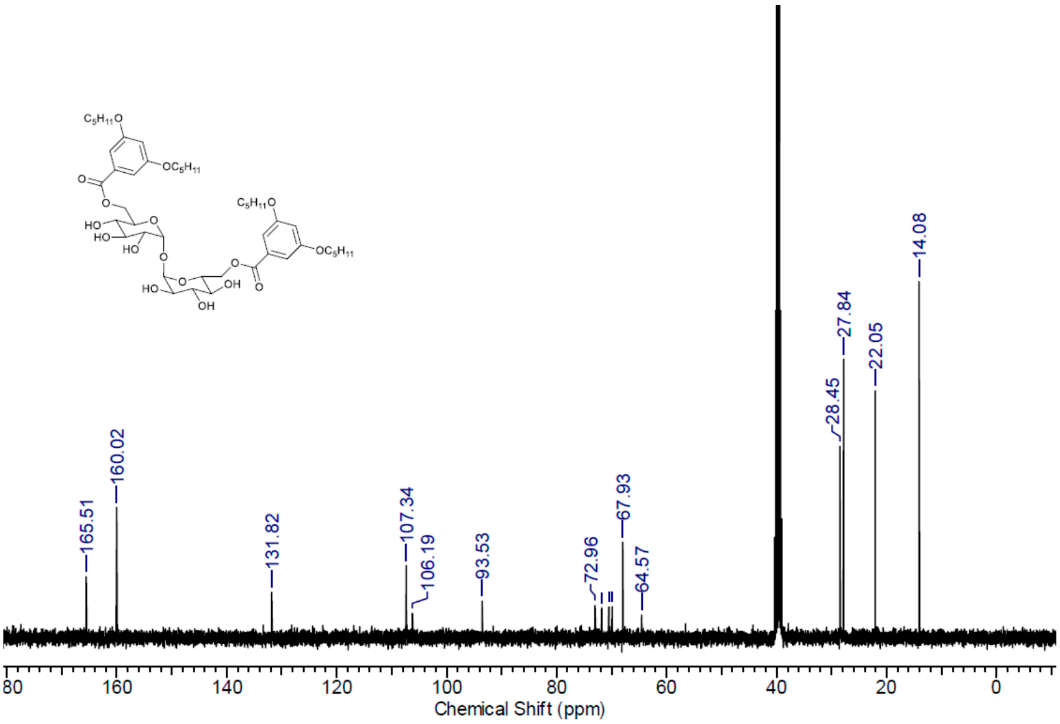

Figure S7: 6,6′-Bis(3,5-dipentyloxybenzoyl)-α,α-D-trehalose (**3e**): ^{1}H NMR (400 MHz, DMSO-D_6) and ^{13}C NMR (101 MHz, DMSO-D_6)

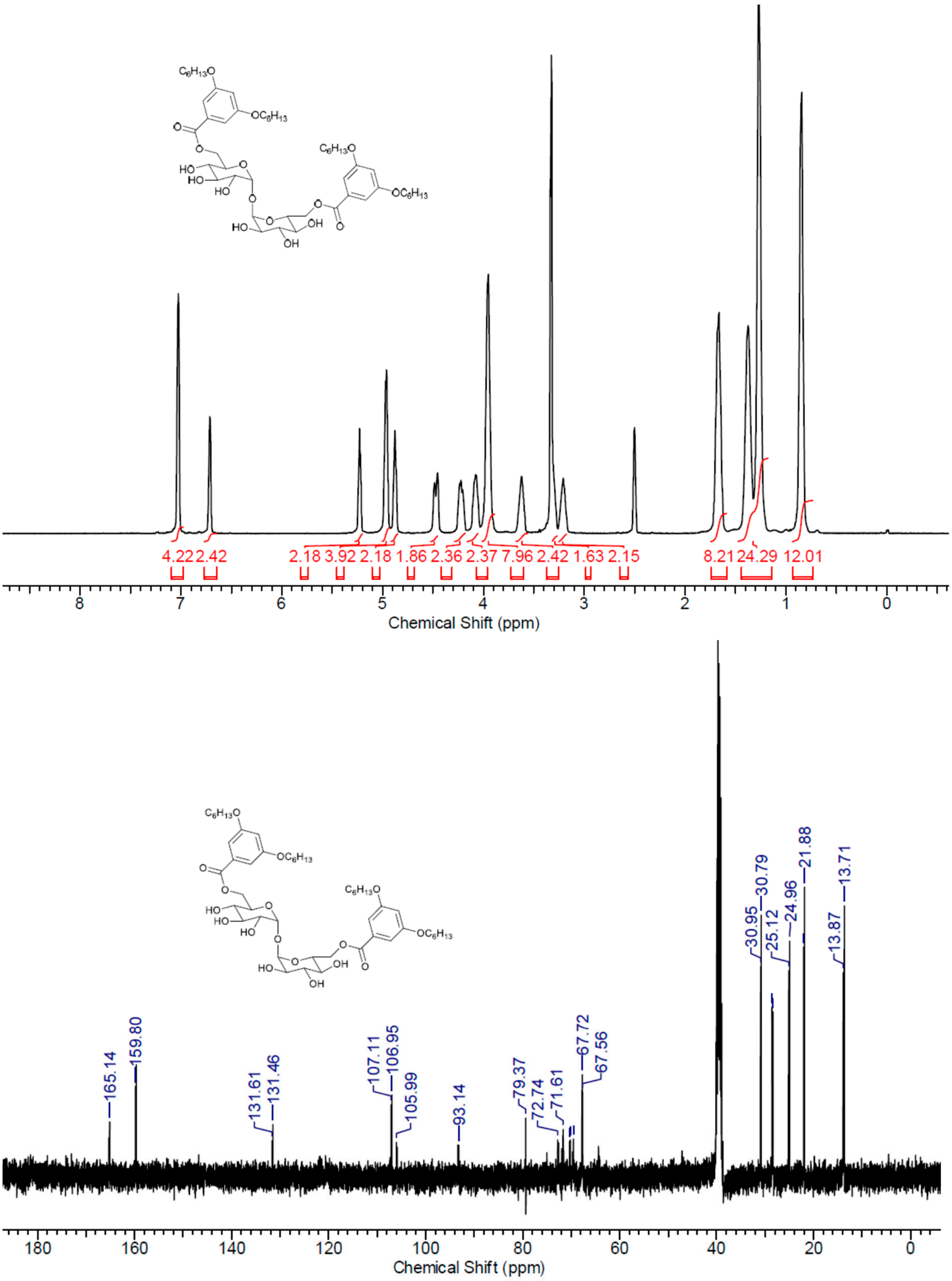

Figure S8: 6,6´-Bis(3,5-dihexyloxybenzoyl)-α,α-D-trehalose (**3f**): ^{1}H NMR (400 MHz, DMSO-D$_6$) and ^{13}C NMR (101 MHz, DMSO-D$_6$).

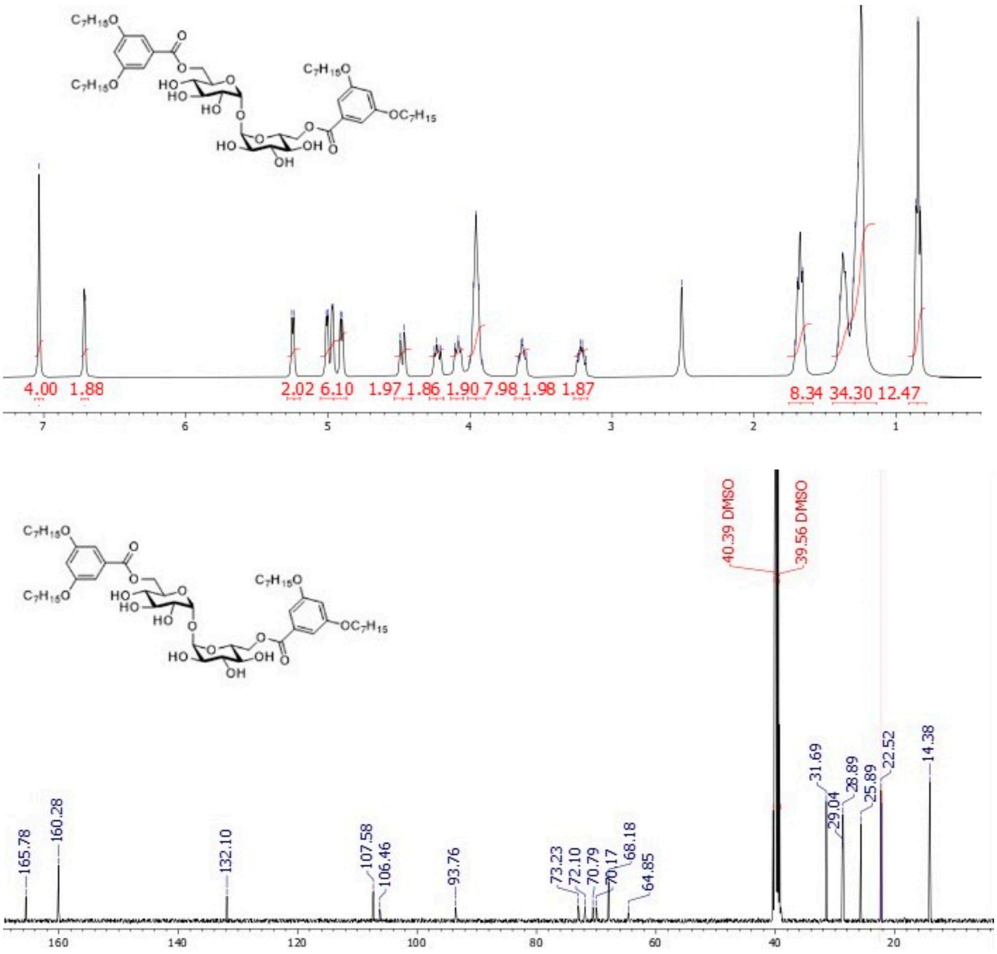

Figure S9: 6,6′-Bis(3,5-diheptyloxybenzoyl)-α,α-D-trehalose (**3g**): ^{1}H NMR (400 MHz, DMSO-D$_6$) and ^{13}C NMR (101 MHz, DMSO-D$_6$).

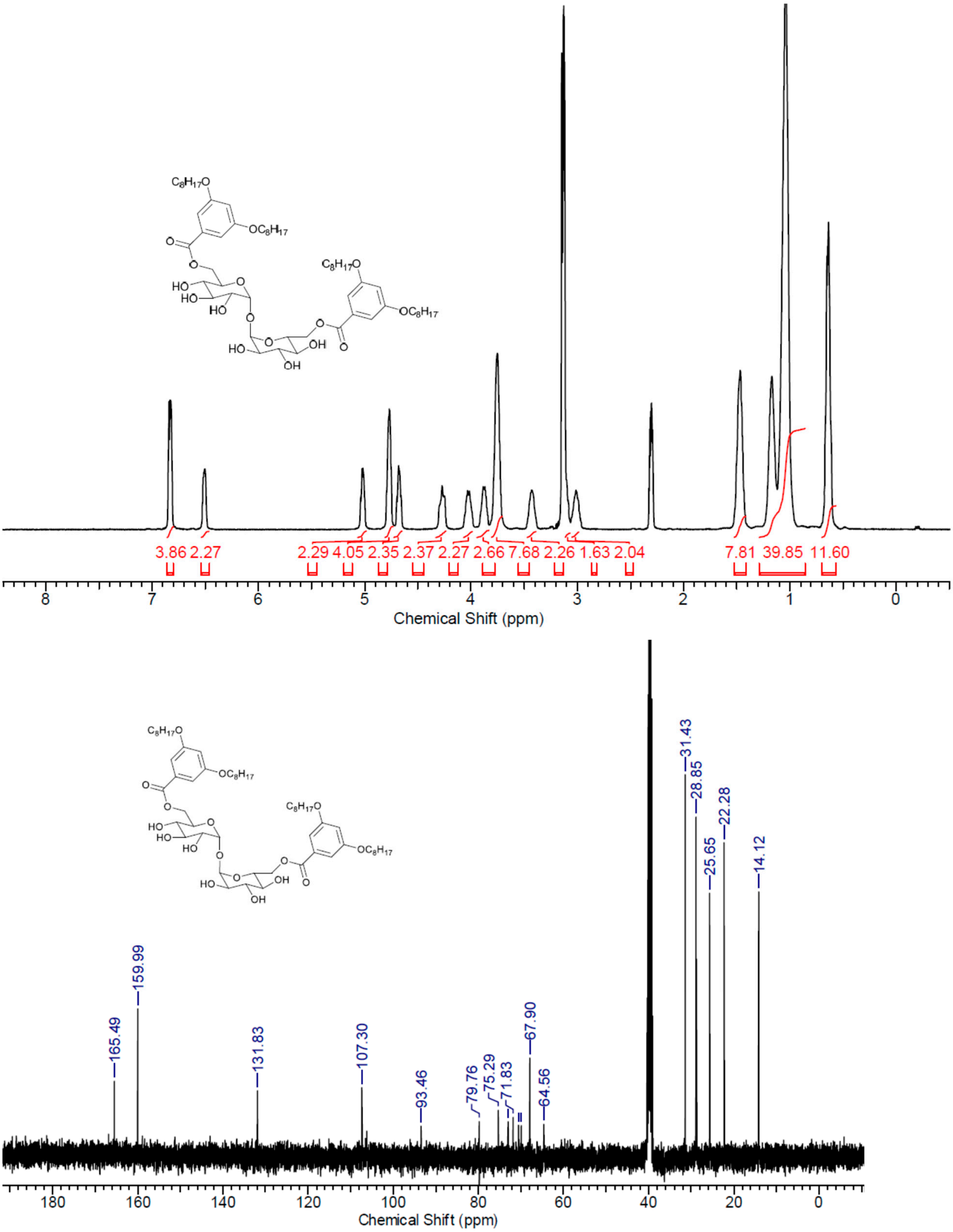

Figure S10: 6,6′-Bis(3,5-dioctyloxybenzoyl)-α,α-D-trehalose (**3h**): ^{1}H NMR (400 MHz, DMSO-D$_6$) and ^{13}C NMR (101 MHz, DMSO-D$_6$).

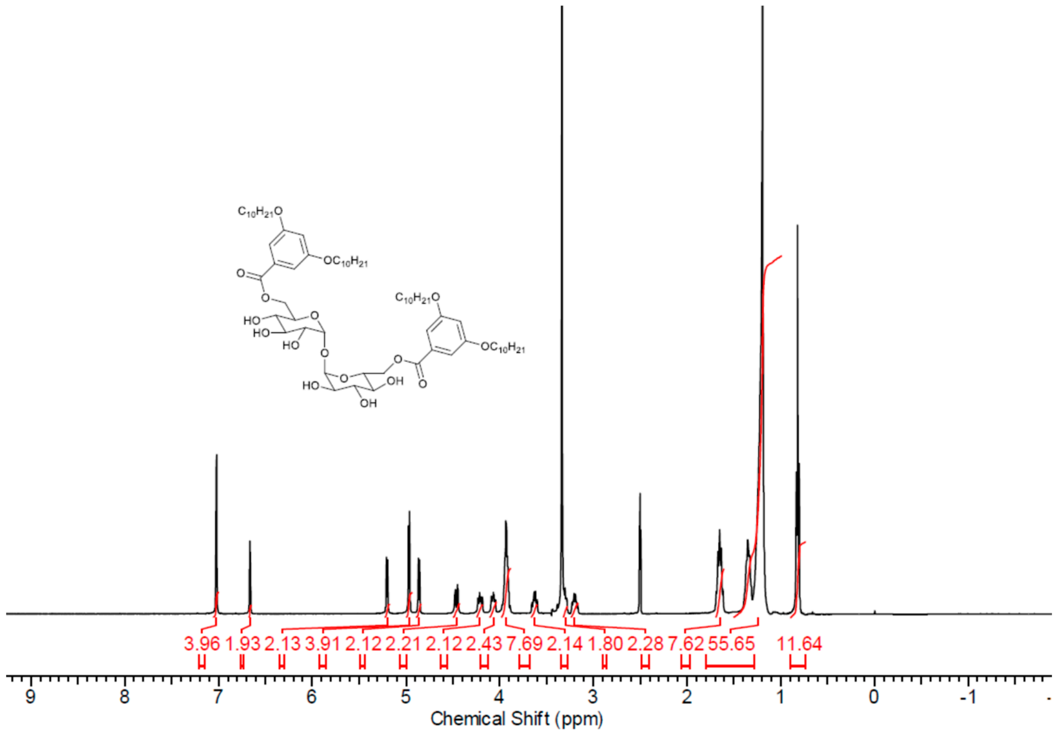

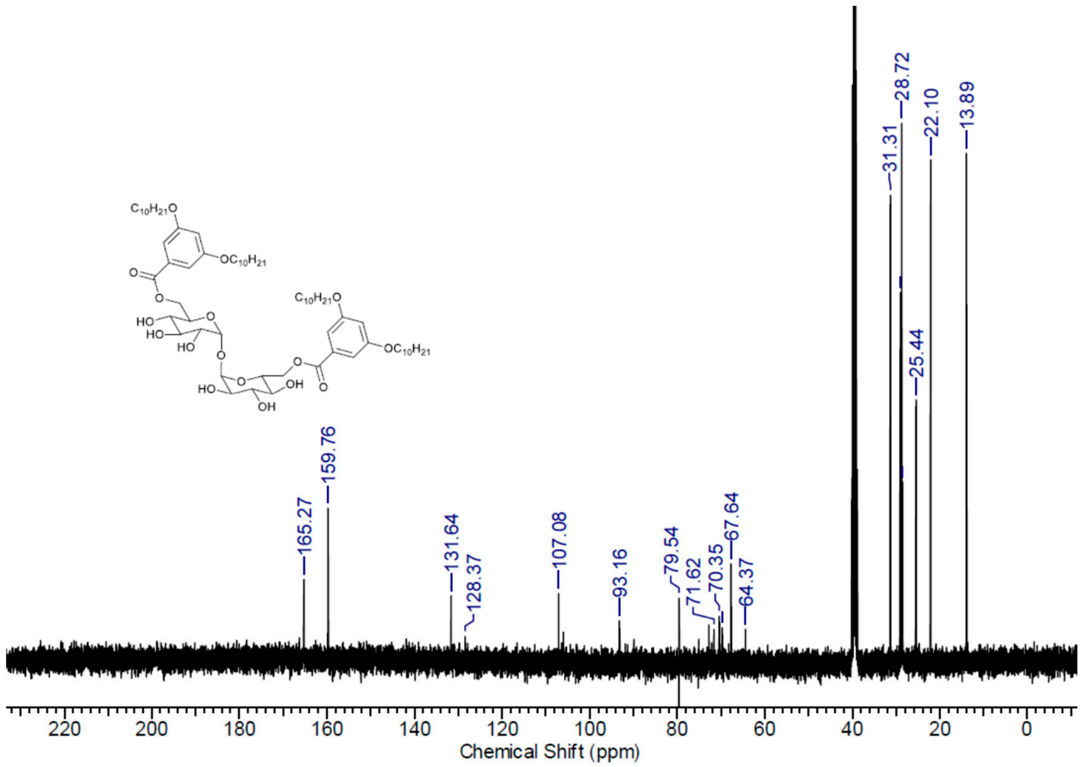

Figure S11: 6,6´-Bis(3,5-didecylyloxybenzoyl)-α,α-D-trehalose (**3i**): ^{1}H NMR (400 MHz, DMSO-D$_6$) and ^{13}C NMR (101 MHz, DMSO-D$_6$).

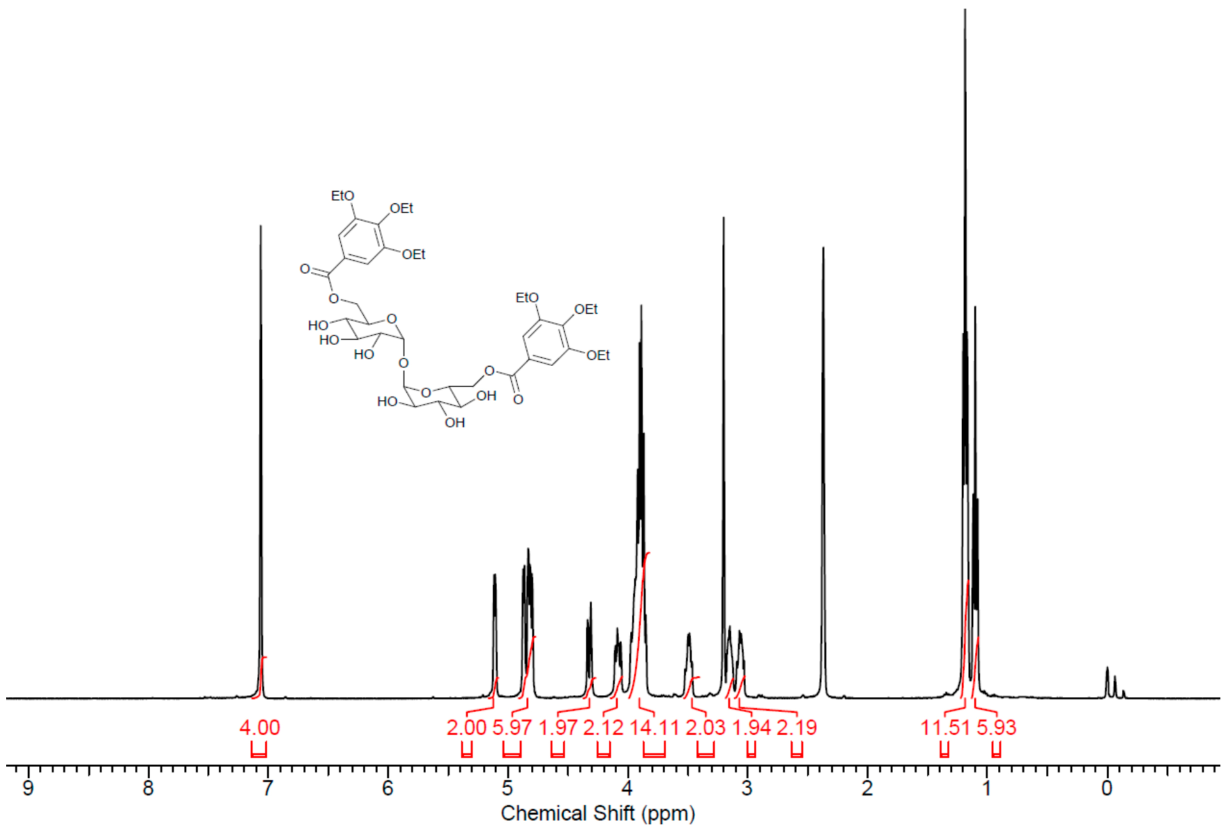

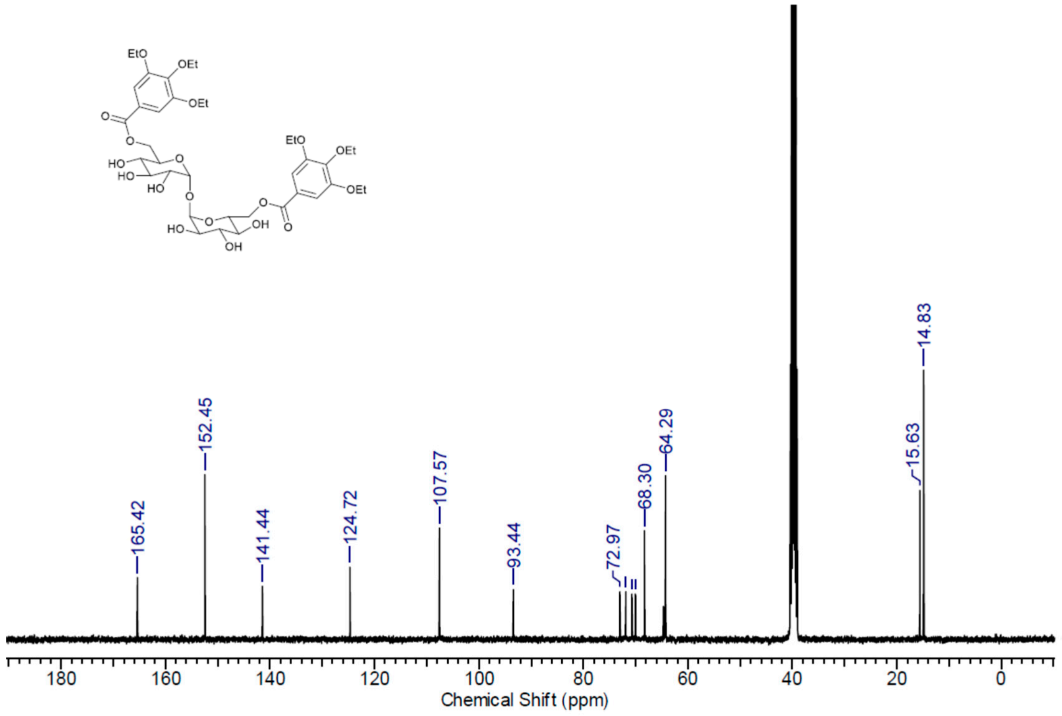

Figure S12: 6,6´-Bis(3,4,5-triethoxybenzoyl)-α,α-D-trehalose (**4a**): ^{1}H NMR (400 MHz, DMSO-D_6) and ^{13}C NMR (101 MHz, DMSO-D_6).

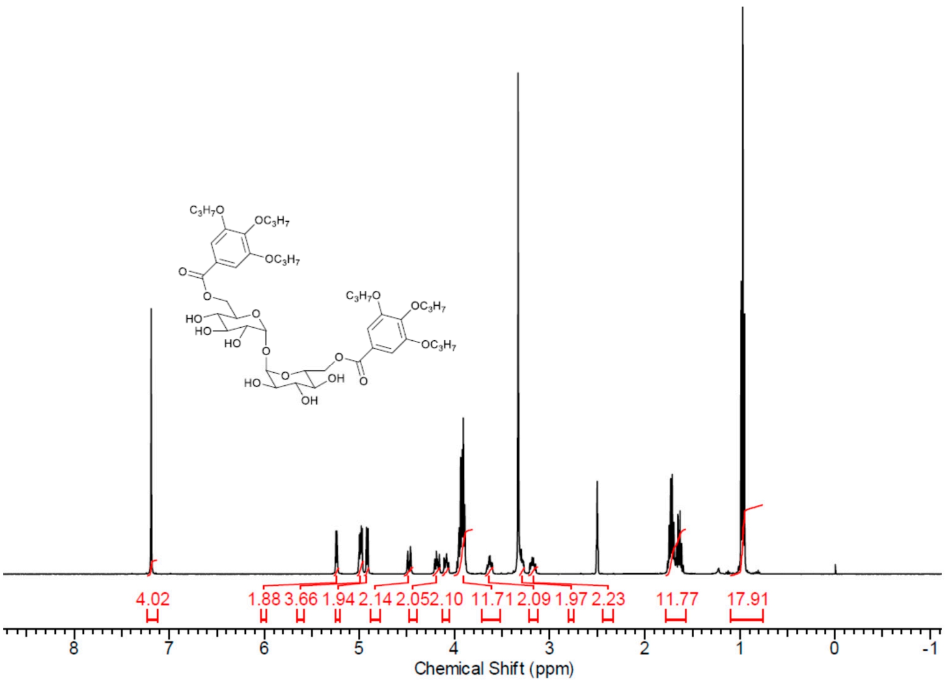

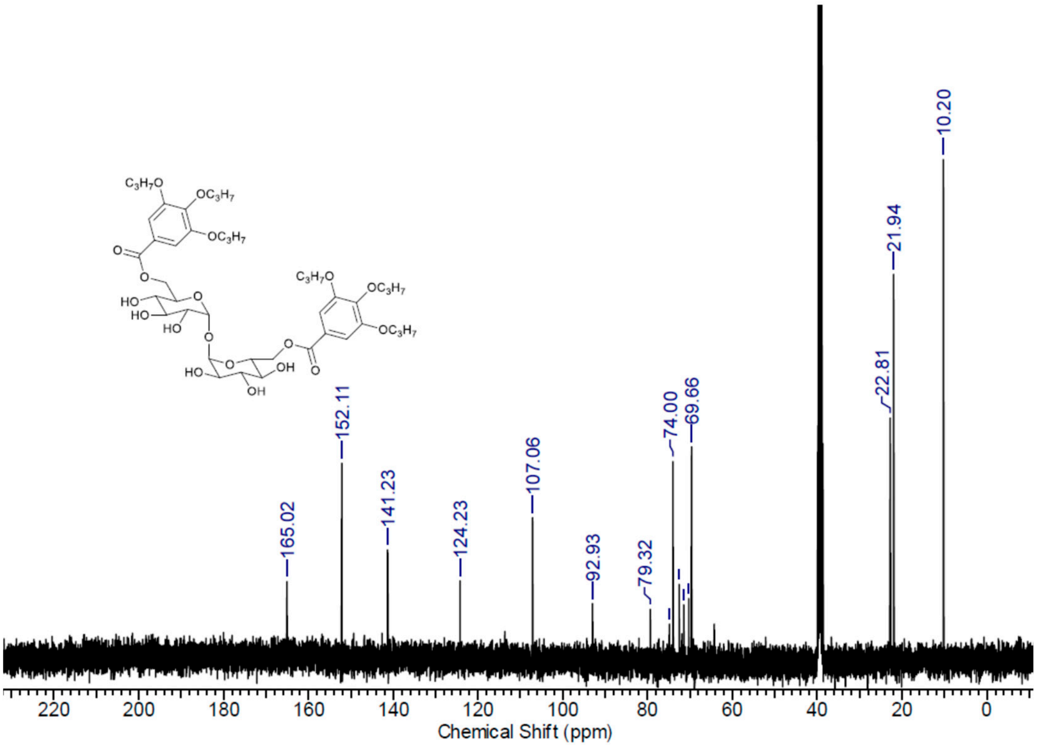

Figure S13: 6,6´-Bis(3,4,5-tripropoxybenzoyl)-α,α-D-trehalose (**4b**): ^{1}H NMR (400 MHz, DMSO-D_6) and ^{13}C NMR (101 MHz, DMSO-D_6).

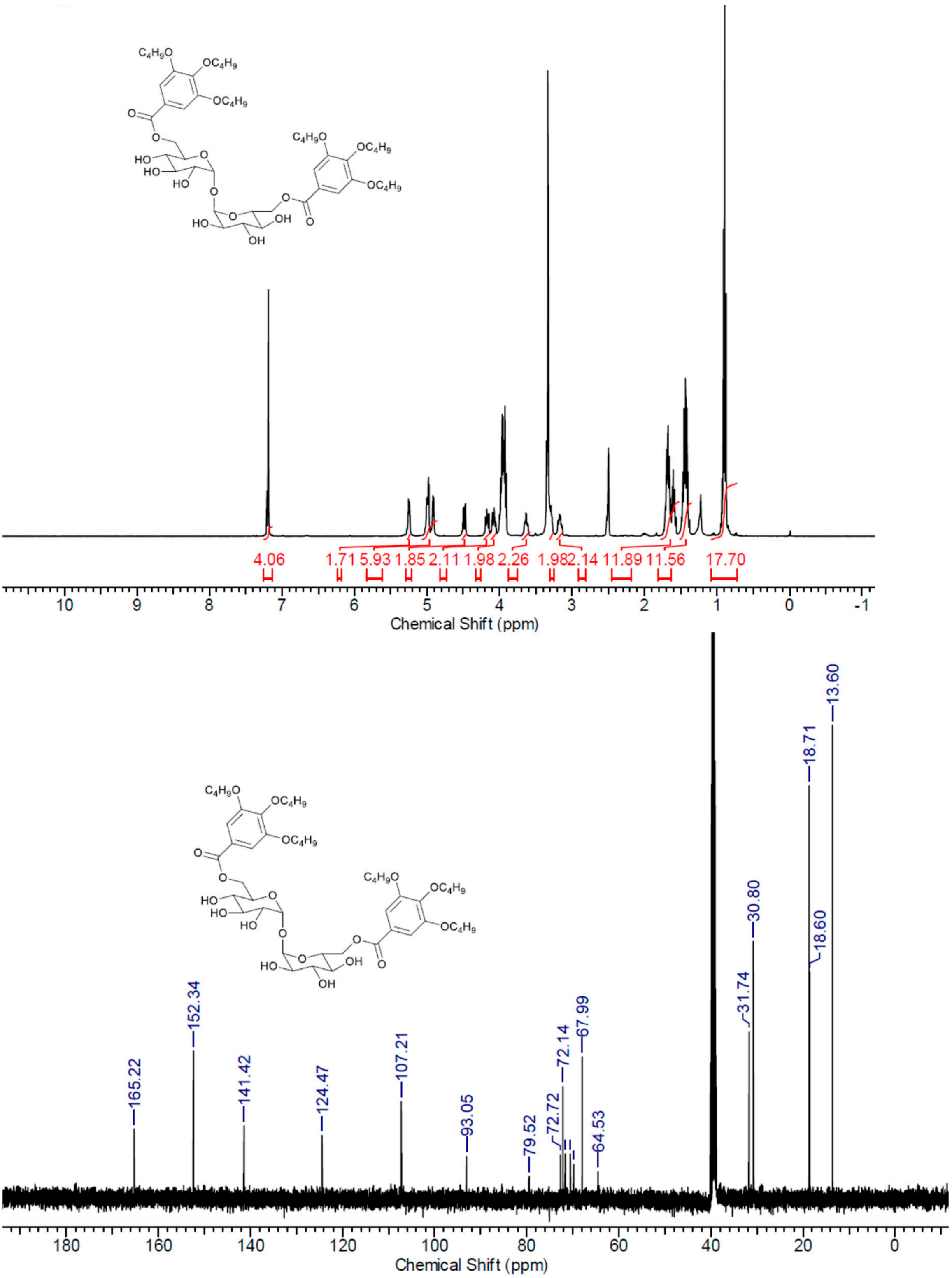

Figure S14: 6,6´-Bis(3,4,5-tributoxybenzoyl)-α,α-D-trehalose (**4c**): ^{1}H NMR (400 MHz, DMSO-D_6) and ^{13}C NMR (101 MHz, DMSO-D_6).

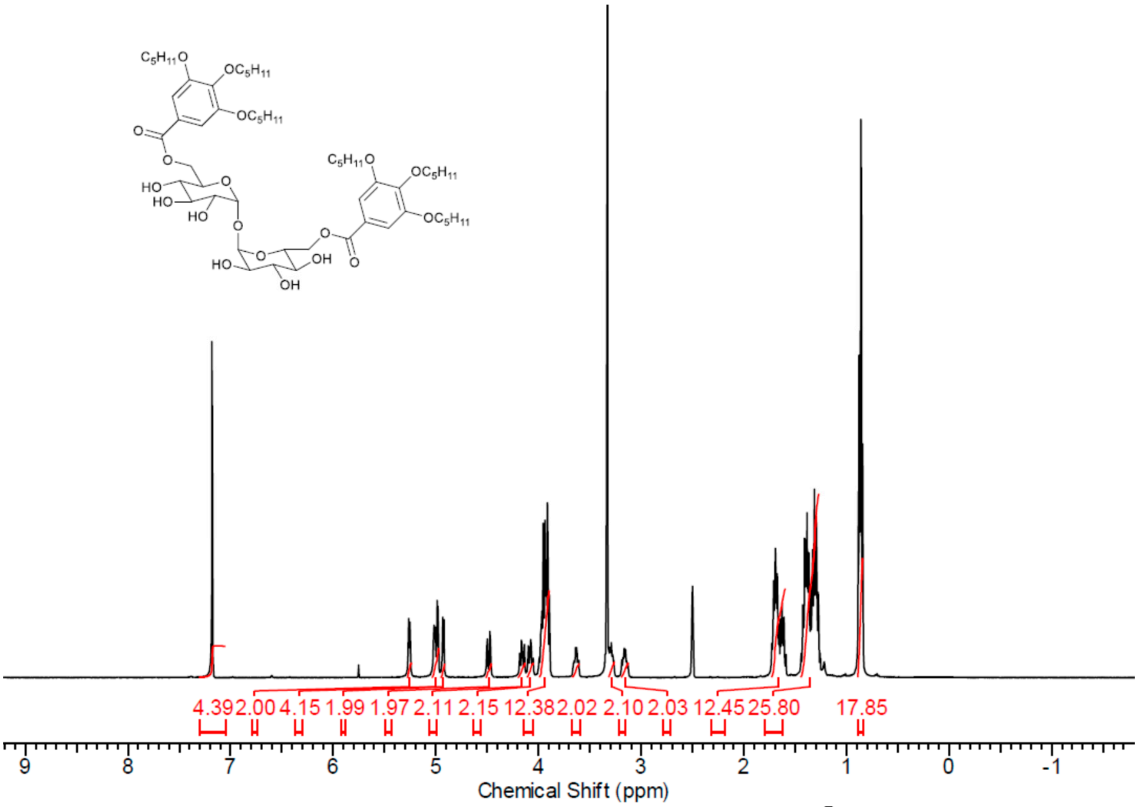

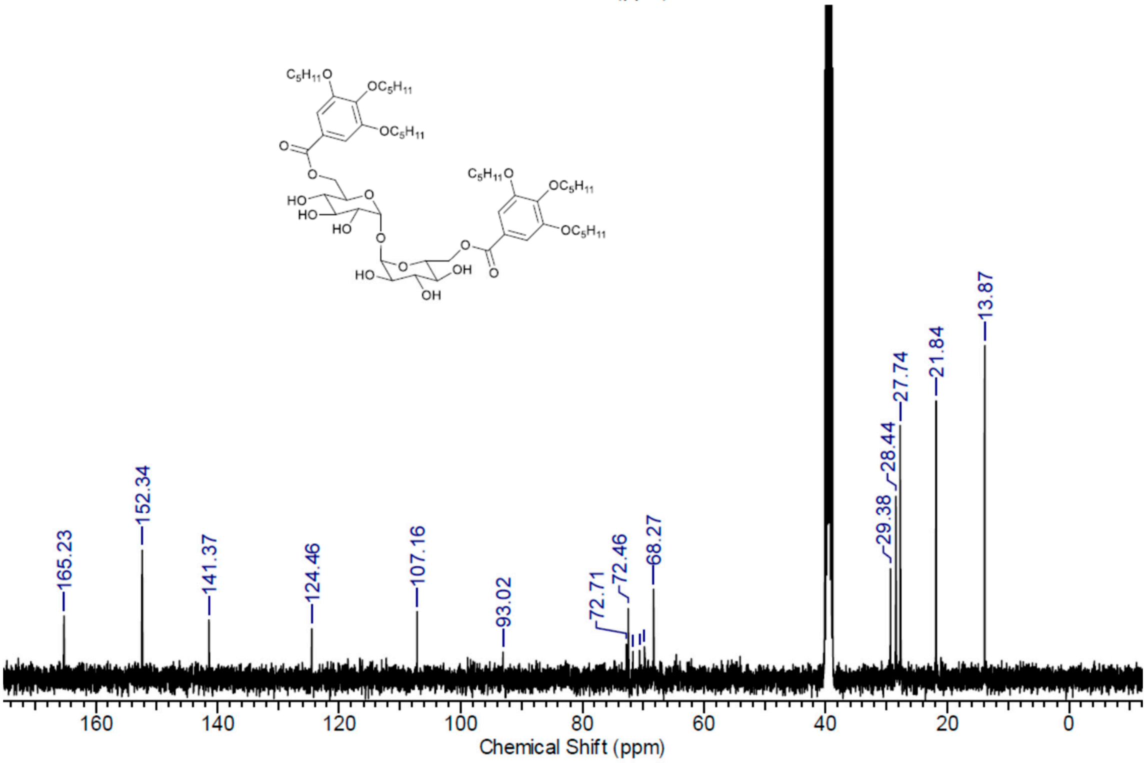

Figure S15: 6,6´-Bis(3,4,5-tripentyloxybenzoyl)-α,α-D-trehalose (**4d**): ^{1}H NMR (400 MHz, DMSO-D$_6$) and ^{13}C NMR (101 MHz, DMSO-D$_6$).

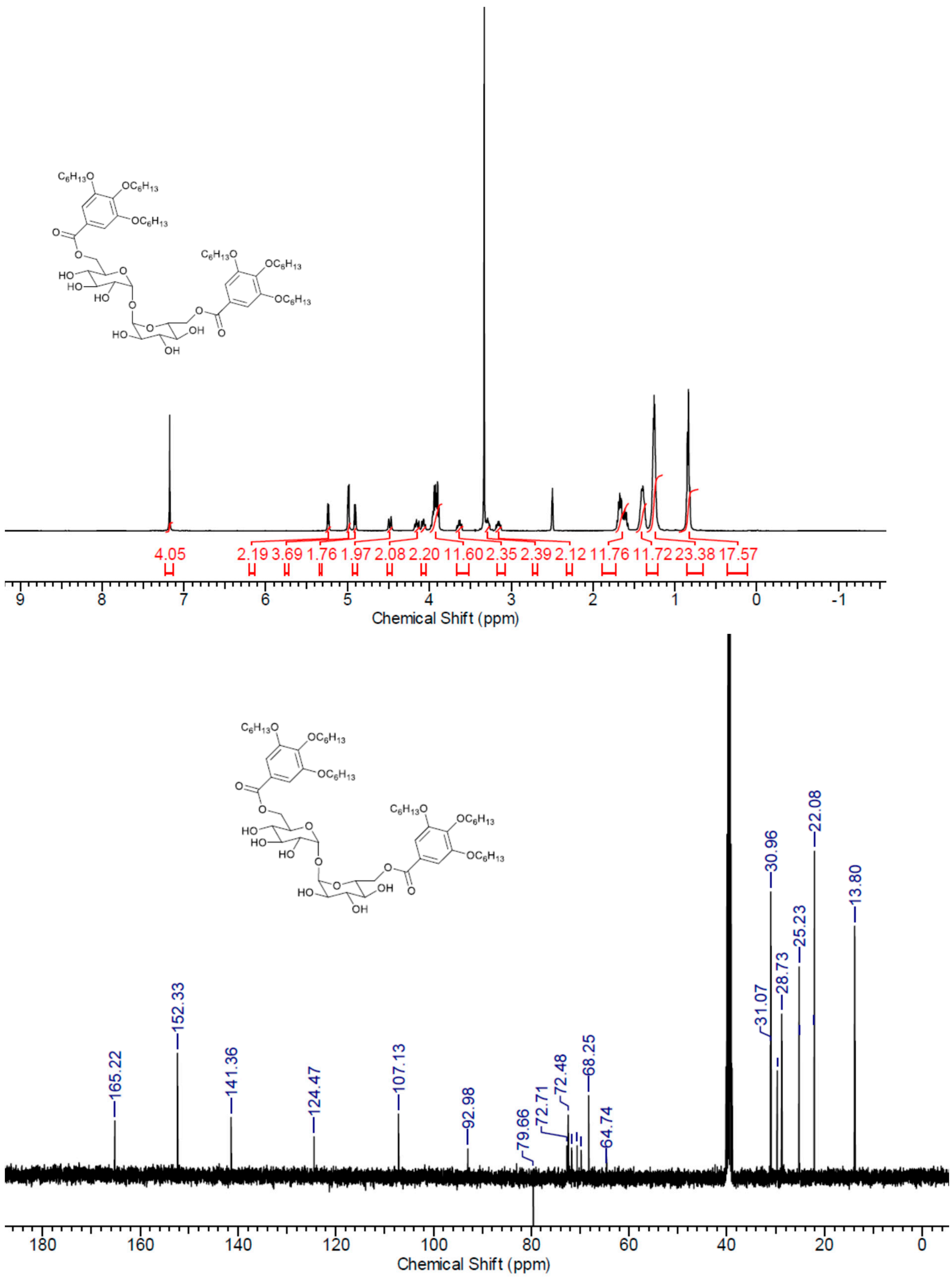

Figure S16: 6,6´-Bis(3,4,5-trihexyloxybenzoyl)-α,α-D-trehalose (**4e**): ^{1}H NMR (400 MHz, DMSO-D$_6$) and ^{13}C NMR (101 MHz, DMSO-D$_6$).

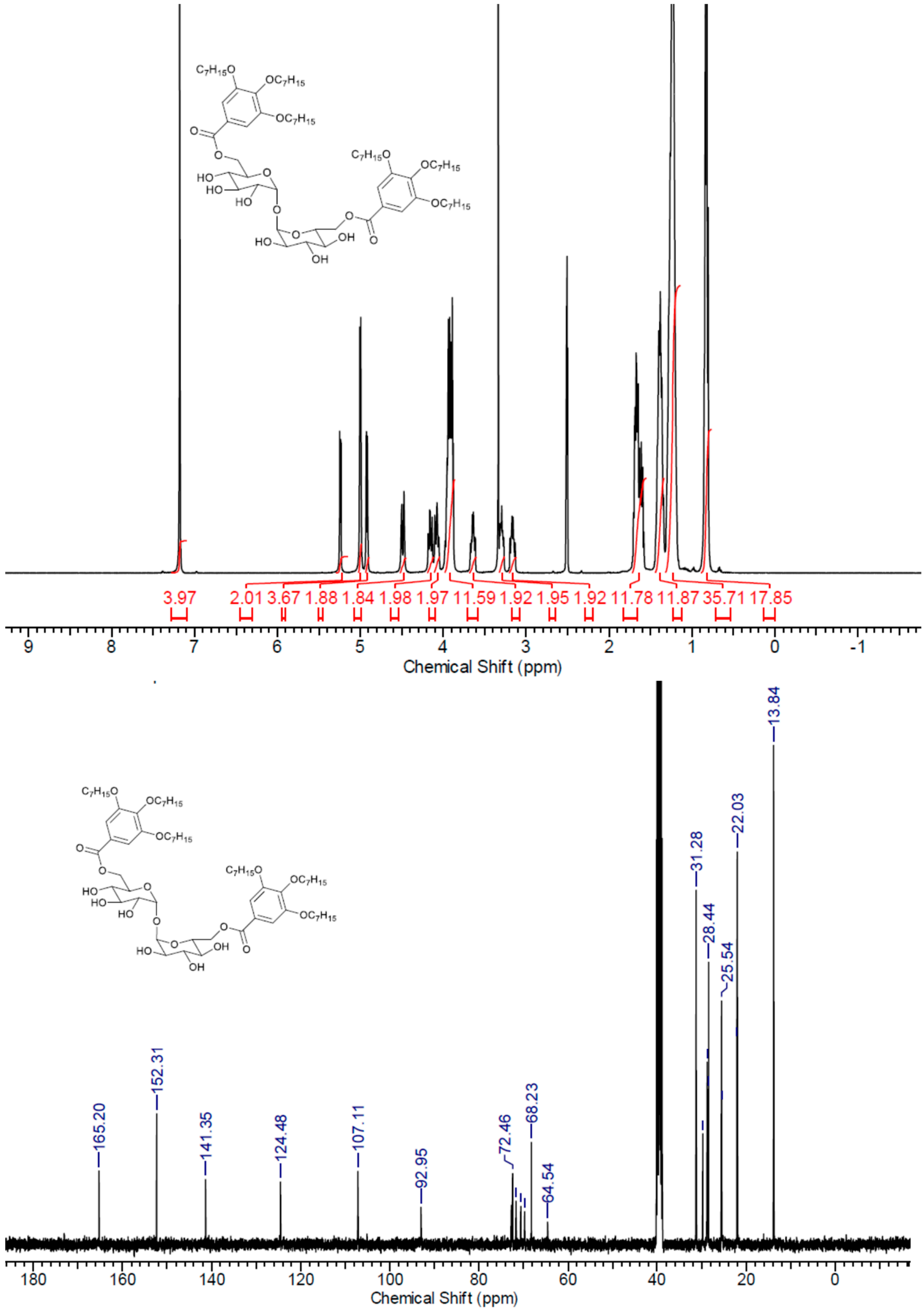

Figure S17: 6,6´-Bis(3,4,5-triheptyloxybenzoyl)-α,α-D-trehalose (**4f**): ^{1}H-NMR (400 MHz, DMSO-D_6) and ^{13}C NMR (101 MHz, DMSO-D_6).

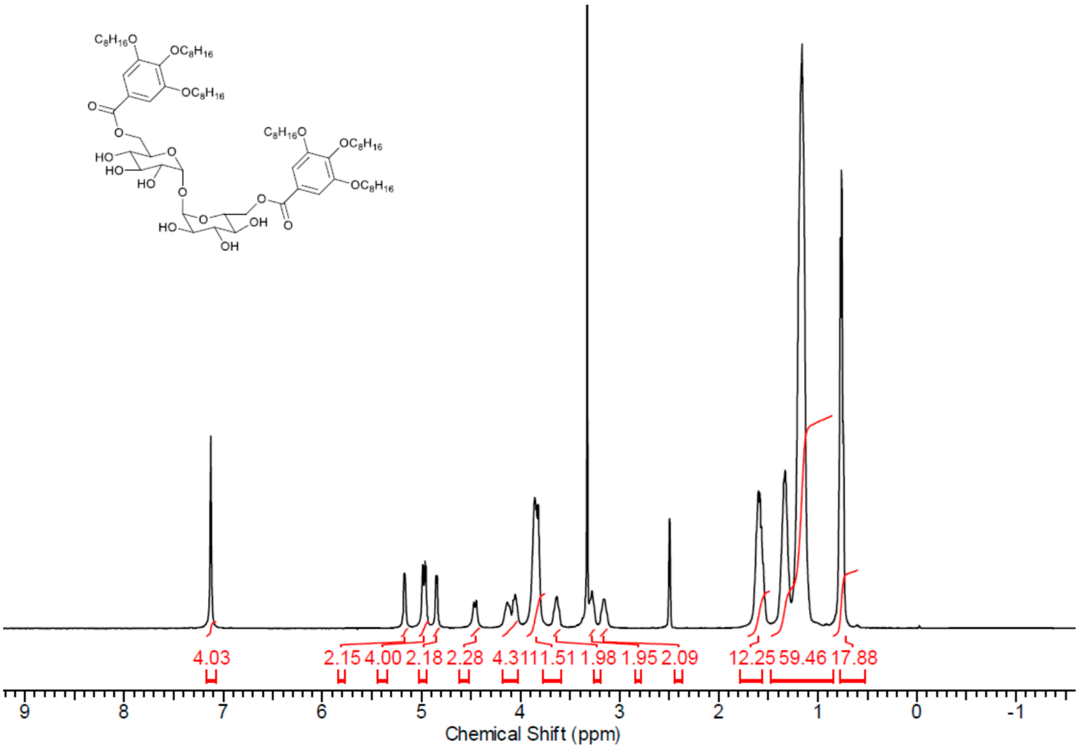

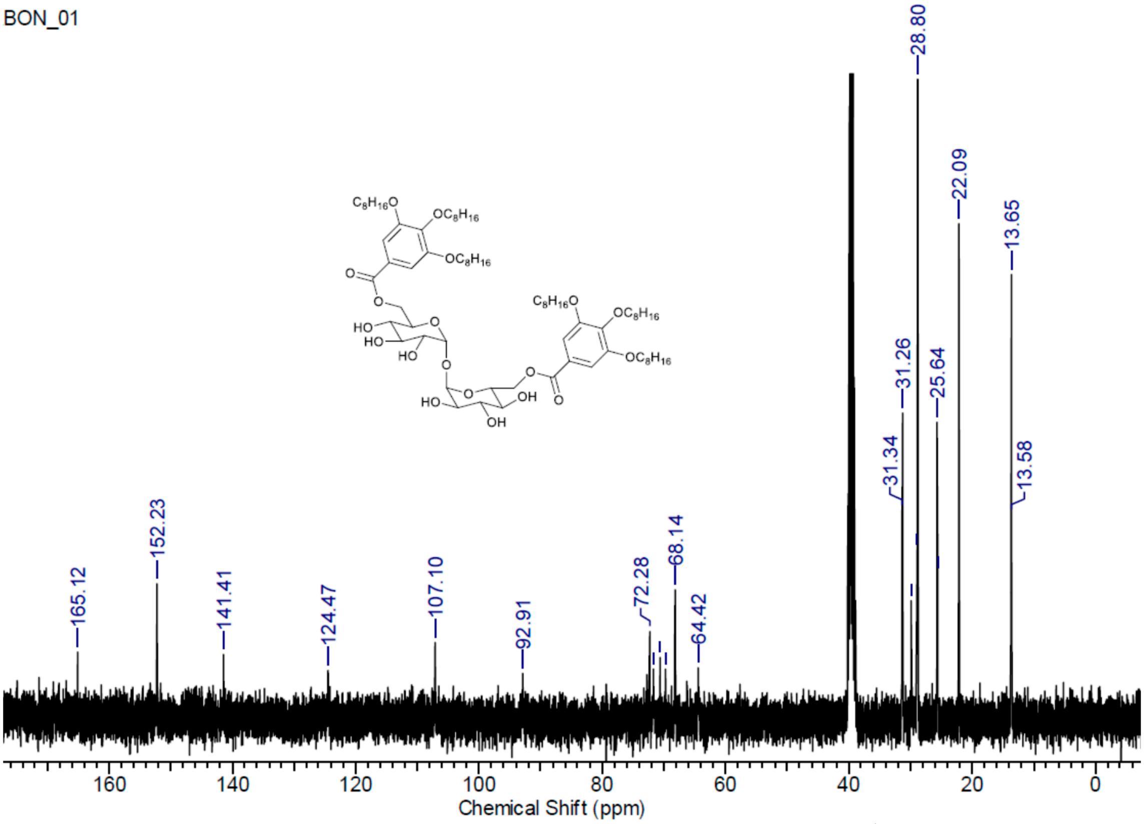

Figure S17: 6,6′-Bis(3,4,5-trioctyloxybenzoyl)-α,α-D-trehalose (**4g**): ^{1}H-NMR (400 MHz, DMSO-D$_6$) and ^{13}C NMR (101 MHz, DMSO-D$_6$).

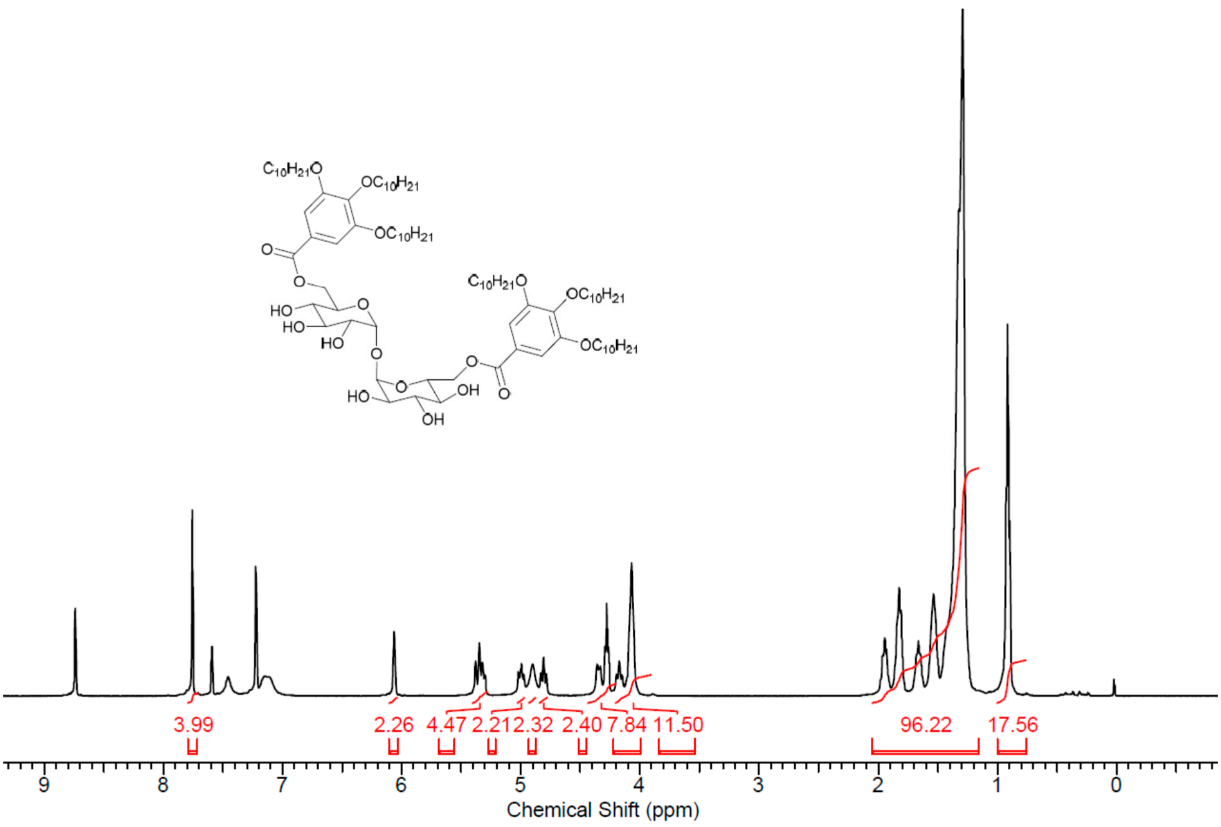

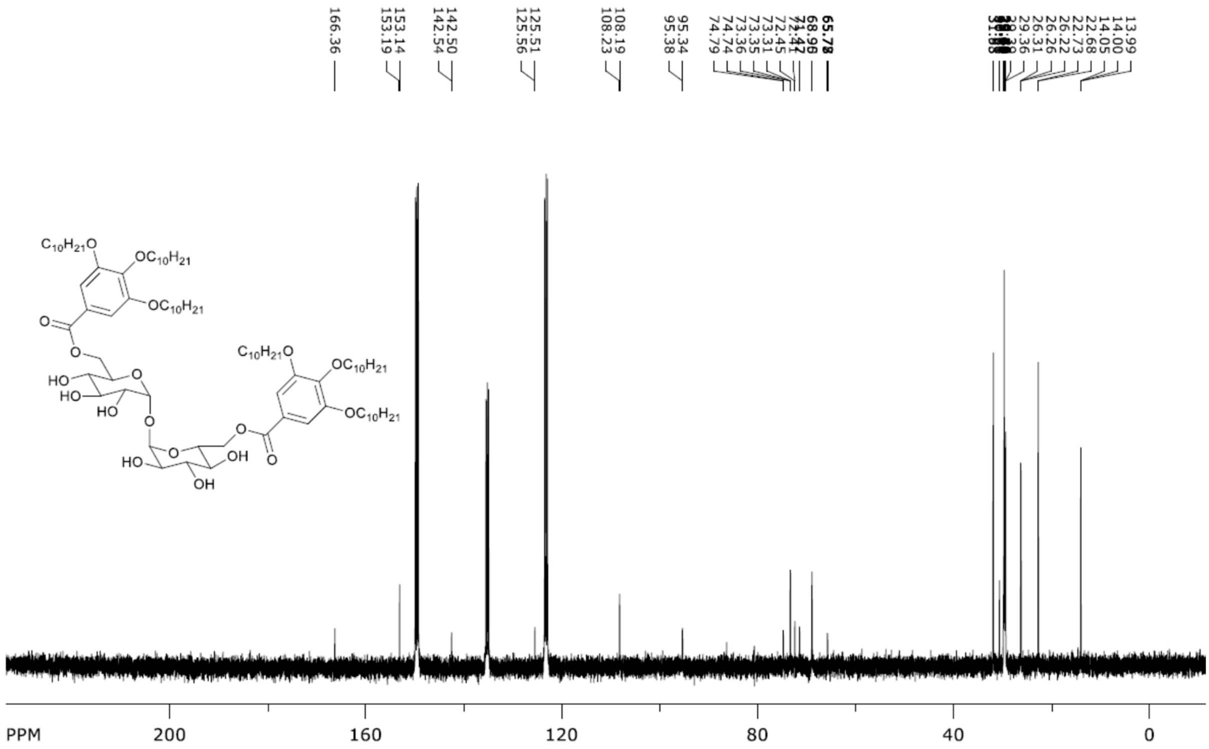

Figure S18: 6,6´-Bis(3,4,5-tridecyloxybenzoyl)-α,α-D-trehalose (**4h**): ^{1}H NMR (400 MHz, Pyridine-D6) and ^{13}C NMR(101 MHz, Pyridine-D6).

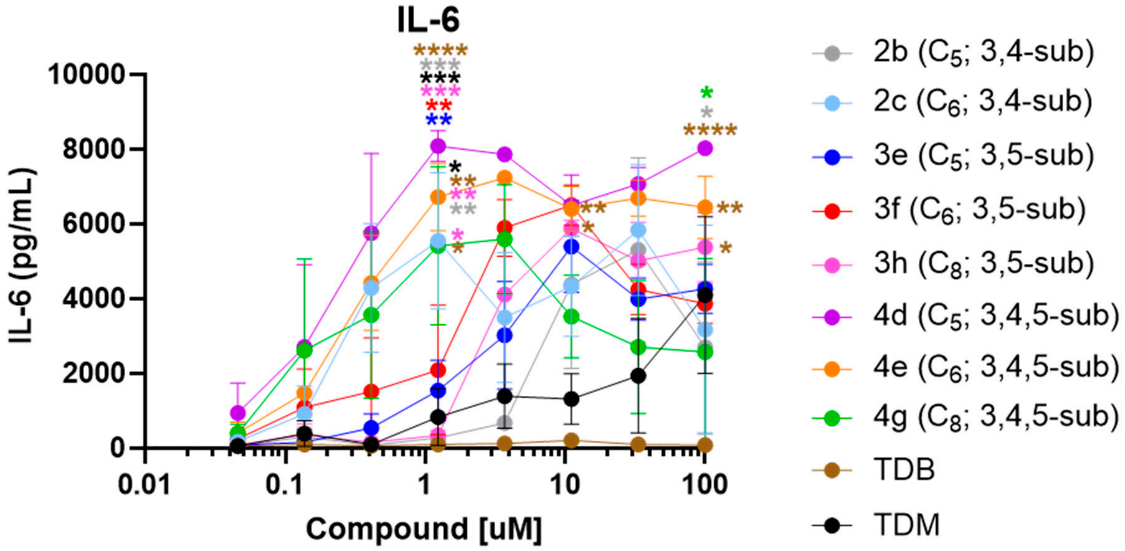

Figure S19. IL-6 production from human PBMCs in response to stimulation with eight trehalose diester compounds. Compounds were dissolved in EtOH, serially diluted in EtOH and then dried to the bottom of a tissue culture plate. Fresh hPBMCs were isolated and added to the compound-coated plates and incubated at 37 °C for 24 hours. Supernatants were harvested and analyzed for IL-6 via MesoScale Discovery multiplex cytokine assay (n = 3 donors). Data was analyzed for statistical significance using 2way ANOVA with Tukey's multiple comparisons test. Significances were considered as follows: ns = $p > 0.05$, * $p \leq 0.05$, ** $p \leq 0.01$, ***$p \leq 0.001$, ****, $p \leq 0.0001$. Significant difference between compounds are indicated at given concentrations. The color of the asterisk above or to the right of a data point indicates to wich color-matched compound a significant difference was measured. Of note, only significant differences at 100, 11.1 and 1.2 µM are shown for graphic simplicity.

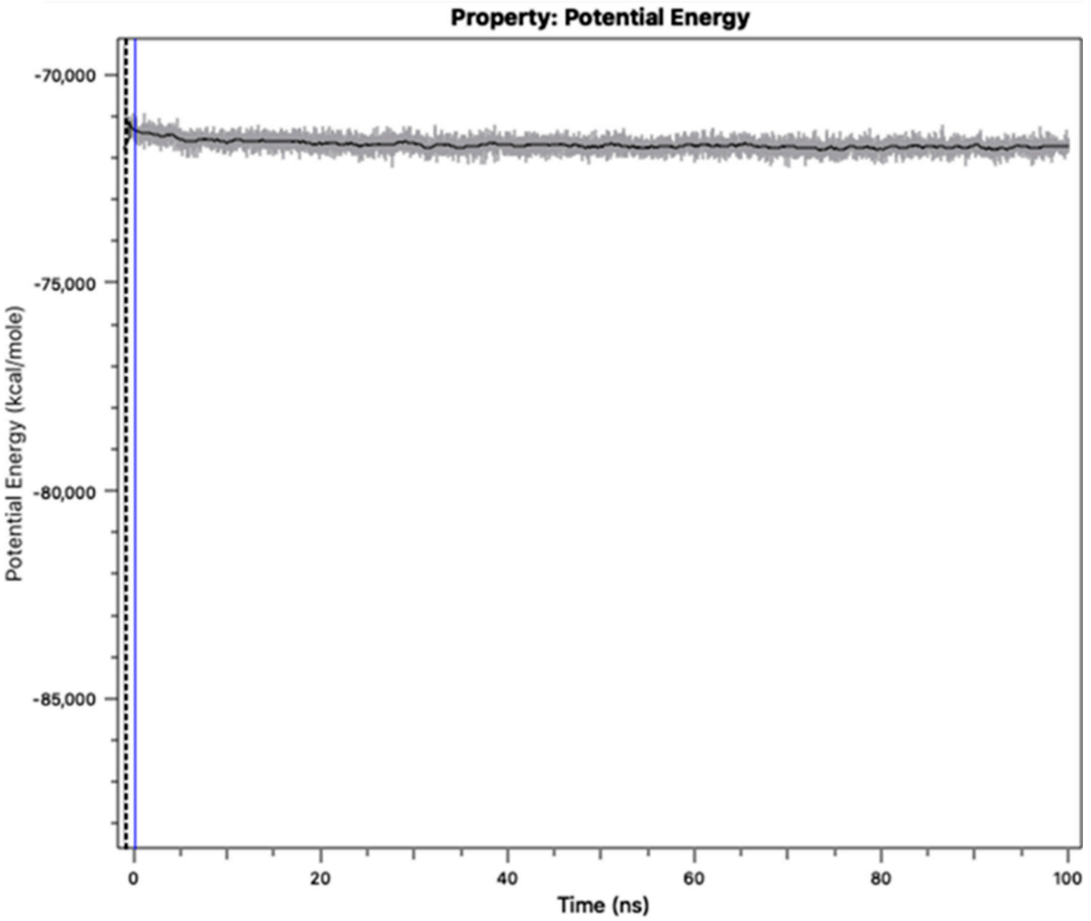

Figure S20. Potential energy plot for each frame of the simulation, overtime. The grey line represents the raw data points while the black line represents the moving average.
